# Epithelial TRIM27 Inhibits Intestinal Inflammation in Ulcerative Colitis by the USP7/TRIM27‐IKK Double Negative‐Feedback

**DOI:** 10.1002/advs.202522114

**Published:** 2026-04-22

**Authors:** Weimin Xu, Zhebin Hua, Zhujiang Dai, Shasha Zhang, Yihan Jiang, Wensong Ge, YingWei Chen, Zhongchuan Wang, Bing Zhang, Chen‐Ying Liu, Peng Du

**Affiliations:** ^1^ Department of Colorectal Surgery, Xinhua Hospital Shanghai Jiaotong University School of Medicine Shanghai China; ^2^ Shanghai Colorectal Cancer Research Center Shanghai China; ^3^ Key Laboratory of Systems Biomedicine Shanghai Center for Systems Biomedicine Shanghai China; ^4^ Department of Cardiovascular Surgery Shanghai Chest Hospital Shanghai China; ^5^ Techniques and Instruments for Diagnosis and Treatment of Congenital Heart Disease Institute of Developmental and Regenerative Medicine Xinhua Hospital Shanghai China; ^6^ Keck School of Medicine University of Southern California Los Angeles California USA; ^7^ Department of Gastroenterology, Xinhua Hospital Shanghai Jiaotong University School of Medicine Shanghai China

**Keywords:** IKK, intestinal inflammation, TRIM27, USP7, ulcerative colitis

## Abstract

The E3 ubiquitin ligase tripartite motif 27 (TRIM27) is a negative regulator of NF‐κB activation and the innate immune response, and TRIM27 deficiency significantly impairs dextran sulfate sodium (DSS)‐induced colitis. The function of TRIM27 in intestinal epithelial cells (IECs), the mechanism by which TRIM27 inhibits the NF‐κB pathway and its dysregulation in ulcerative colitis (UC) remain unclear. Here, it is report that epithelial TRIM27 functions as an anti‐inflammatory factor that inhibited intestinal inflammation in IECs in vitro and in epithelial *Trim27* knockout mice in vivo. Mechanistically, TRIM27 destabilized IKKα and TRAF6 via polyubiquitination of IKKα at the K569 site and TRAF6 at the K489 site. In response to TNF‐α, IKKβ phosphorylated TRIM27 at S173 to decrease TRIM27 expression by impairing its binding to ubiquitin‐specific protease 7 (USP7) and USP7‐mediated TRIM27 deubiquitination. Notably, overexpression of TRIM27 enhanced the anti‐inflammatory effect of infliximab (IFX) in IECs. TRIM27 is downregulated in inflamed colons from UC patients and is associated with the therapeutic effect of IFX. Overall, this study identifies epithelial TRIM27 as a bona fide negative modulator of intestinal inflammation and USP7/TRIM27‐IKK as a new double negative feedback mechanism of the NF‐κB pathway, which supports the use of TRIM27 replenishment as a potential therapeutic strategy for UC.

## Graphical abstract

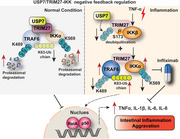

## 1 Introduction

Ulcerative colitis (UC) is a chronic inflammatory bowel disease (IBD) characterized by inflammation of the colorectal mucosa, crypt abscess of the intestinal mucosa, and decreased or absent secretion of intestinal epithelial mucin [1]. In contrast to transmural inflammation in Crohn's disease (CD), the other type of IBD, inflammation in UC is typically limited to the mucosal layer [1]. UC is an intestinal barrier disease driven initially by dysregulated intestinal epithelial cells (IECs) and disrupted intestinal barrier [1]. Therefore, the inflammatory response of the intestinal epithelium and the abnormally activated inflammatory cascade in IECs are the core factors in the pathogenesis of UC [2]. Currently, although biologics, such as anti‐tumor necrosis factor (TNF‐α), are widely used and show promising efficacy in the treatment of UC, a considerable proportion of patients fail to achieve sustained clinical remission and mucosal healing [3]. Nearly 20% of UC patients require surgical intervention due to drug therapy failure or serious complications [3], which further highlights the complexity of UC etiology and the urgent clinical need for new therapies. A deep understanding of the epithelial pathways in intestinal homeostasis and inflammation is vital for the development of new targeted therapies to promote epithelial repair and mucosal healing in UC [4, 5].

Tripartite motif (TRIM) family proteins, most of which have E3 ubiquitin ligase activities due to their RING‐finger domain, mediate the ubiquitination of numerous protein substrates to regulate diverse biological processes, including inflammatory diseases and carcinogenesis [6]. Tripartite motif‐containing 27 (TRIM27), also known as Ret finger protein (RFP), functions as a RING‐mediated E3 ubiquitin ligase to induce the ubiquitination of various substrates, such as NOD2, PI3KC2β, TAK1 binding protein 2/3 and P53 [7, 8, 9]. By downregulating TAB2/3 and P53, TRIM27 attenuates the inflammatory response and protects against liver and cardiac ischemia/reperfusion injury [7, 8]. TRIM27 is also involved in the pathogenesis and progression of IBD [9, 10]. In addition to acting as a negative regulator of the NOD2‐mediated inflammatory response, a previous study reported the negative regulation of NF‐κB activation and innate immune response by TRIM27 via interactions with IκB kinase family members [11]. However, TRIM27 expression is increased in the colon of CD patients and in the CD4^+^ T lymphocytes of mesenteric lymph nodes in a DSS‐induced colitis model, indicating a proinflammatory role of TRIM27 in colitis [9, 12]. Knockout of *Trim27* significantly diminishes dextran sulfate sodium (DSS)‐induced STAT3 activation and colitis in *Trim27* ^−/−^ conventional knockout mice [10]. Reciprocal bone marrow chimera experiments have revealed that TRIM27 deficiency in hematopoietic cells is responsible for the attenuation of DSS‐induced colitis [10]. Recently, TRIM27 was identified as a stabilizer of β‐catenin that promotes intestinal stem cell (ISC) self‐renewal and maintains gut homeostasis [13]. The *Trim27* ^−/−^ conventional knockout mice showed dysfunction of Lgr5+ ISCs and spontaneous irritable bowel syndrome (IBS)‐like symptoms [13]. The gene expression of TRIM27 is known to be downregulated in IBS patients; however, the dysregulation of TRIM27 in UC and the function and regulatory mechanisms of TRIM27 in the inflammatory response in IECs remain largely undefined.

In the present study, we identified TRIM27 as a negative regulator of intestinal inflammation in IECs by establishing *Villin‐Trim27^flox/flox^* mice. TRIM27 inhibited intestinal epithelial inflammation by promoting the ubiquitination and subsequent degradation of TNF receptor‐associated factor 6 (TRAF6) and IκB kinase α (IKKα). Importantly, IKKβ phosphorylated TRIM27 at S173, which suppressed its interaction with ubiquitin‐specific protease 7 (USP7) and USP7‐mediated TRIM27 deubiquitination, leading to decreased TRIM27 protein abundance in response to TNF‐α stimulation. Thus, our study reveals a double negative feedback loop of the IKK complex involving TRIM27 and the anti‐inflammatory role of TRIM27 in IECs, highlighting the therapeutic potential of TRIM27 re‐expression in UC.

## 2 Results

### 2.1 TRIM27 inhibits Intestinal Epithelial Inflammation

**FIGURE 1:**
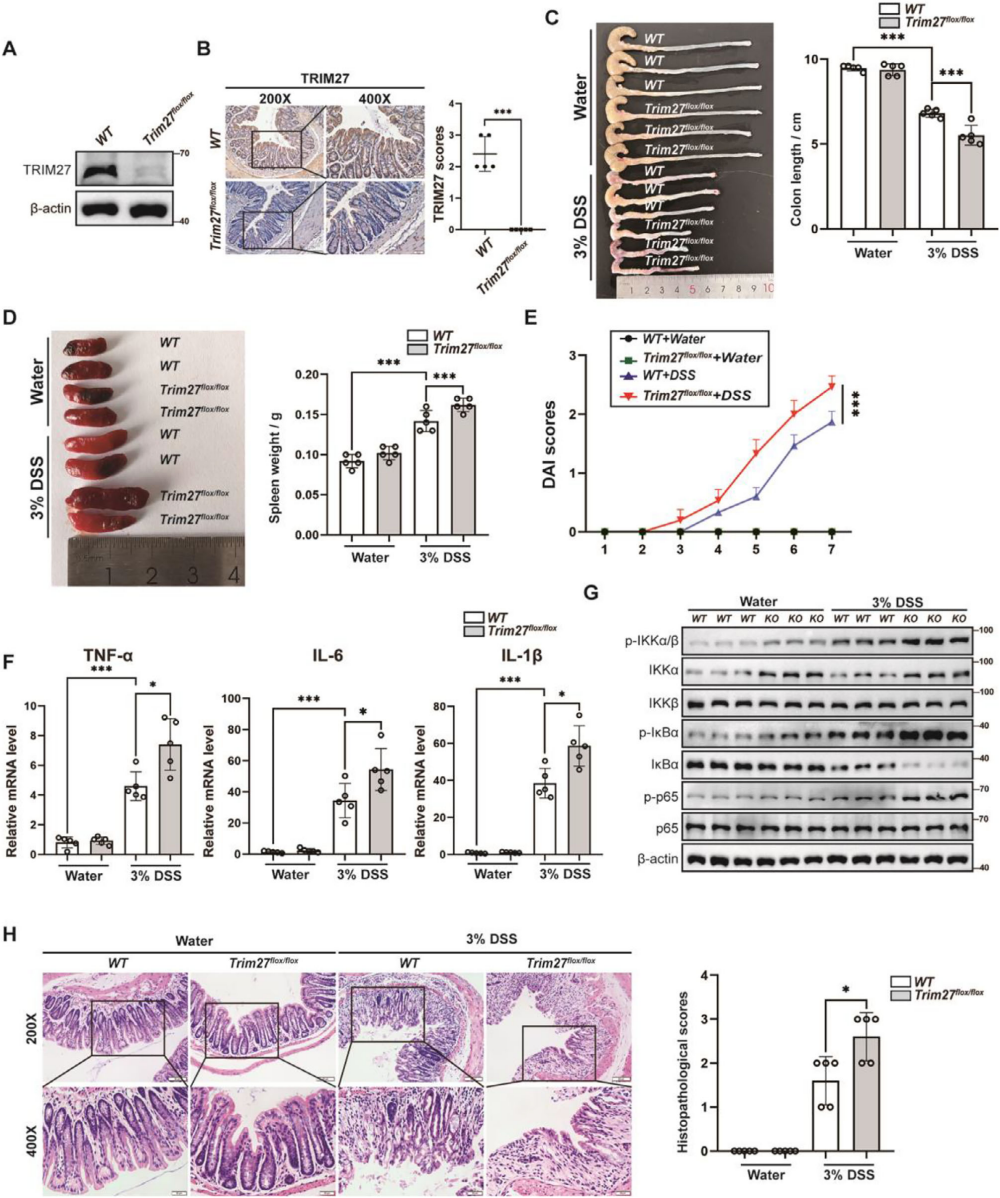
TRIM27 alleviates DSS‐induced intestinal mucosal inflammation in mice. (A,B) The efficiency of TRIM27 knockdown in intestinal epithelium from *WT* and *Trim27^flox/flox^* mice was examined by western blot analysis and IHC. (C,D) Representative images indicating the colorectum lengths and spleen weights in control and DSS‐treated *WT* and *Trim27^flox/flox^* mice (*n* = 5 for each water group, *n* = 5 for each DSS group). (E) The loss of body weight and the severity of diarrhoea and bleeding, as evaluated by the DAI score, were determined in control and DSS‐treated *WT* and *Trim27^flox/flox^* mice. (F–H) The expression of proinflammatory cytokines (TNF‐α, IL‐6, IL‐1β), phosphorylation‐mediated activation of the NF‐κB pathway (p‐IKKα/β, IKKα, IKKβ, p‐IκBα, IκBα, p‐p65, and p65) and the pathological scores in colons from control and DSS‐treated *WT* and *Trim27^flox/flox^* mice were evaluated by qRT‒PCR, western blotting and hematoxylin–eosin staining (magnification: 200×, upper panels; 400×, lower panels), respectively. The replicate WB results are available in the attached original figures. The data are presented as the means ± SDs. Wilcoxon signed‐rank test (B), One‐way ANOVA (C, D, F), two‐way ANOVA (E) and the Mann‒Whitney's U test (H) were performed to assess statistical significance. * *p* < 0.05, *** *p* < 0.001.

To assess the function of TRIM27 in IECs in colitis, we generated *Villin‐Trim27^flox/flox^* mice and confirmed the knockout of *Trim27* by western blot and immunohistochemistry analyses (Figure 1A,B). After 7 consecutive days of DSS treatment, we observed that the *Villin‐Trim27^flox/flox^* mice presented shorter colon lengths, heavier spleen weights and higher DAI scores than their *WT* littermates did (Figure 1C–E). Consistent with the above findings, both the mRNA levels of inflammatory cytokine genes and the activation of the NF‐κB pathway in *Villin‐Trim27^flox/flox^* mice were significantly greater than those in their *WT* littermates (Figure 1F,G). To evaluate the histopathological lesions of the colon, hematoxylin–eosin staining was performed. As shown in Figure 1H, pathological scores were higher in colon tissues *from Villin‐Trim27^flox/flox^* mice than in those from *WT* mice.

Next, we examined intestinal barrier injury in *Villin‐Trim27^flox/flox^* mice and their *WT* littermates in a DSS‐induced colitis model. As shown in Figure S1A, the number of goblet cells in *Villin‐Trim27^flox/flox^* mice was lower than that in their *WT* littermates in the DSS‐treated group but not in the control group, as shown by alcian blue/periodic acid‐Schiff staining, which indicated that *Trim27* knockout aggravated goblet cell destruction in the context of colitis. To strengthen these data, immunofluorescence staining of intestinal epithelial markers was performed and revealed that the expression of ZO‐1, MUC2, E‐cadherin and CK20 was moderately downregulated after inflammatory stimulation in *Villin‐Trim27^flox/flox^* mice compared with their *WT* littermates (Figure S1B–E). These results indicate that TRIM27 significantly attenuated DSS‐induced acute colitis and protected the integrity of the intestinal barrier.

Consistent with the in vivo observations, similar results were observed in the intestinal epithelial cell lines HT29 and HIEC‐6 following overexpression and knockout of TRIM27 (Figures S2A,B and S3A,B). We found that forced expression of TRIM27 significantly inhibited the levels of proinflammatory cytokines (e.g., TNF‐α, IL‐1β, and IL‐8) after TNF‐α stimulation (Figure S2C,D). The phosphorylation of IKKα/β, IκBα, and P65 was markedly decreased in TRIM27‐overexpressing HT29 and HIEC‐6 cells (Figure S2E). In addition, immunofluorescence staining revealed that the overexpression of TRIM27 abrogated the TNF‐α‐induced translocation of p65 from the cytoplasm to the nucleus in HIEC‐6 cells after TNF‐α treatment (Figure S2F). Conversely, the TNF‐α‐induced transcription of these proinflammatory cytokine genes were significantly increased in TRIM27‐deficient cells (Figure S3C,D). Moreover, knockout of TRIM27 in IECs promoted the activation of the IKK complex and its downstream phosphorylation events (Figure S3E). Consistent with the above findings, overexpression of TRIM27 in human colon organoids also significantly inhibited TNF‐α‐induced inflammation (Figure S4A–D). Collectively, these data indicate that TRIM27 inhibits the activation of NF‐κB signaling and plays an anti‐inflammatory role in IECs. The bar graphs showing grayscale value of WB data of Figure 1, Figures S2 and S3 were shown in Supporting files.

### 2.2 TRIM27 Promotes IKKα Degradation by Ubiquitination at Lysine Residue 569

**FIGURE 2:**
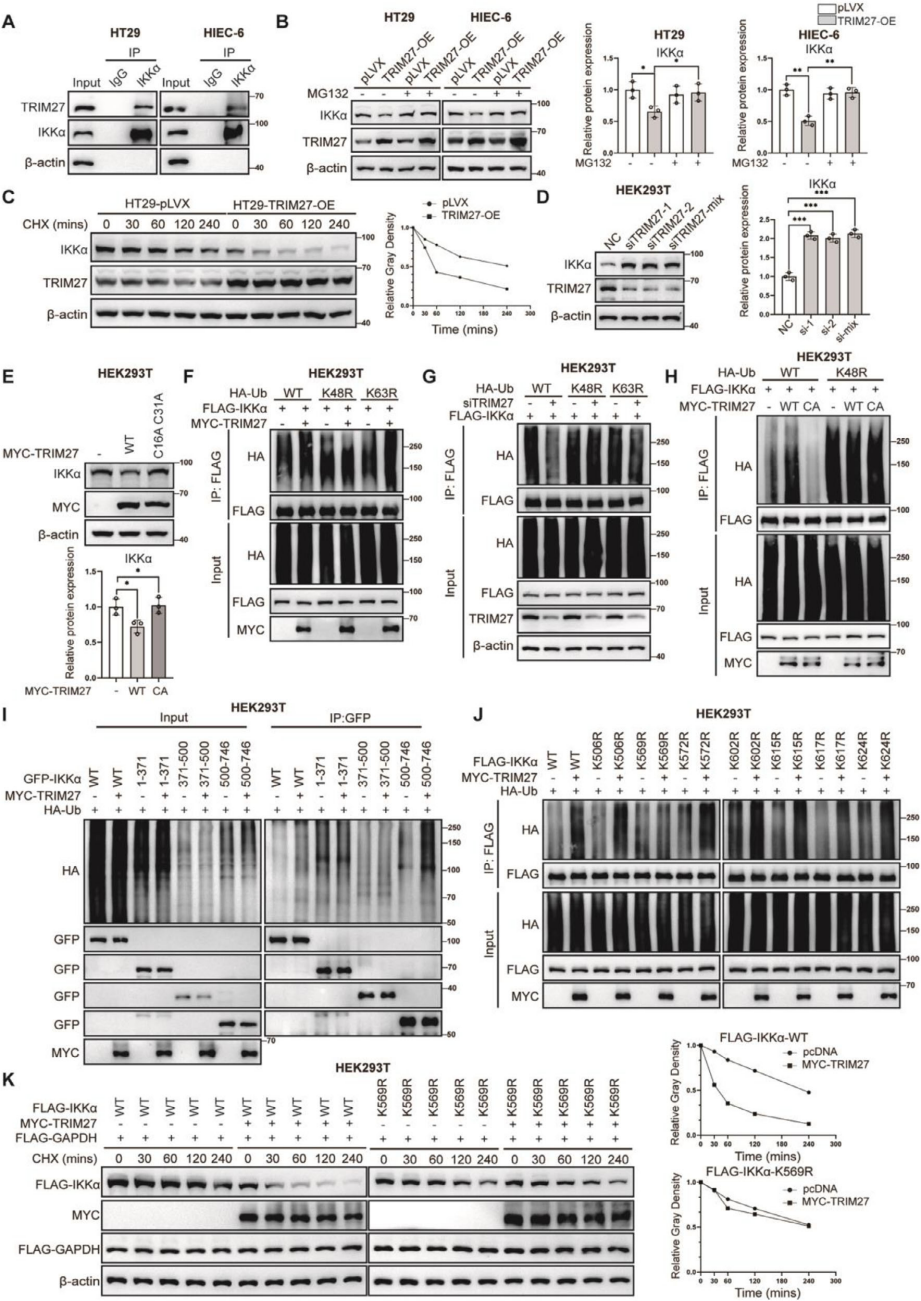
TRIM27 promotes IKKα degradation by ubiquitination at lysine residue 569. (A) endogenous Co‐IP of TRIM27 and IKKα in HT29 and HIEC‐6 cells. (B) The protein levels of IKKα were detected in control and TRIM27‐overexpressing HT29 and HIEC‐6 cells with or without MG132 treatment by western blots. (C) Analysis of the IKKα protein half‐life in control and TRIM27‐overexpressing HT29 cells. Cells were treated with cycloheximide (CHX) for the indicated times before western blot analysis of IKKα expression. (D) The IKKα protein levels were examined in HEK293T cells transfected with control and siRNA targeting TRIM27 by western blots. (E) HEK293T cells were transfected with the WT and the E3 ligase dominant negative mutant (C16A& C31A) of MYC‐TRIM27. IKKα expression was examined by western blots. (F) HEK293T cells were transfected with HA‐Ub, HA‐Ub‐K48R, HA‐Ub‐K63R, FLAG‐IKKα, and MYC‐TRIM27. Cell lysates were used for immunoprecipitation of FLAG‐IKKα and subsequent quantification of the ubiquitination level by western blots. (G) The control and TRIM27‐delepted HEK293T cells were transfected with HA‐Ub, HA‐Ub‐K48R, HA‐Ub‐K63R, and FLAG‐IKKα. FLAG‐IKKα was immunoprecipitated for subsequent quantification of the ubiquitination level of IKKα by western blots. (H) HEK293T cells were transfected with HA‐Ub, HA‐Ub‐K48R, FLAG‐IKKα, MYC‐TRIM27, and MYC‐TRIM27^C16A&C31A^. The ubiquitination level of IKKα was analyzed as described above. (I) HEK293T cells were transfected with HA‐Ub, full‐length and 1–371, 371–500, 500–746 truncation of GFP‐IKKα with or without coexpression of MYC‐TRIM27. Cell lysates were used for immunoprecipitation of GFP‐IKKα and subsequent quantification of the ubiquitination level via western blotting. (J) HEK293T cells were transfected with HA‐Ub, FLAG‐IKKα^WT^, FLAG‐ IKKα^K506R^, FLAG‐IKKα^K569R^, FLAG‐IKKα^K572R^, FLAG‐IKKα^K602R^, FLAG‐IKKα^K615R^, FLAG‐IKKα^K617R^, FLAG‐IKKα^K624R^ and MYC‐TRIM27. Cell lysates were used for immunoprecipitation of FLAG‐IKKα and subsequent quantification of the ubiquitination level via western blotting. (K) Analysis of the half‐life of the WT and K569R mutant FLAG‐IKKα in HEK293T cells transfected with control and MYC‐TRIM27 plasmids. Cells were treated with CHX for the indicated times before western blot analysis. One‐way ANOVA (B, D, E) was performed to assess statistical significance. * *p* < 0.05, ** *p* < 0.01, *** *p* < 0.001.

A previous study identified TRIM27 as an IKK‐interacting protein in a yeast two‐hybrid screen and subsequently revealed that TRIM27 can also interact with TBK1 and IKKα/β to suppress IFN‐stimulated response elements (ISREs) and NF‐κB activation [11]. Intriguingly, we observed that TRIM27 overexpression markedly reduced the protein level of IKKα in HT29 and HIEC‐6 cells, and the protein level of IKKα was mildly increased in the colons of *Villin‐Trim27^flox/flox^* mice (Figure 1G; Figure S2E). These data suggest that TRIM27 may negatively influence the protein stability of IKKα in IECs. To clarify the mechanism by which TRIM27 downregulates the IKKα protein, we first confirmed the interaction between TRIM27 and IKKα by exogenous and semiendogenous co‐IP in HEK293T cells (Figure S5A,B). The interactions between endogenous TRIM27 and IKKα were further confirmed in HT29 and HIEC‐6 cells (Figure 2A). To explore the nature of the interaction between TRIM27 and IKKα, we generated various truncated forms of TRIM27 and found that only the N‐terminal RING‐finger (1‐62) and coiled coil (133‐295) domains mediated the interaction with IKKα (Figure S5C). As expected, the decrease in the IKKα protein level induced by TRIM27 overexpression was abrogated by treatment with the proteasome inhibitor MG132 (Figure 2B). A cycloheximide (CHX) assay further revealed that TRIM27 overexpression decreased the half‐life of the endogenous IKKα protein in HT29 and HIEC‐6 cells (Figure 2C; Figure S5D). Furthermore, we found that TRIM27 knockdown significantly increased the protein level of IKKα (Figure 2D). To strengthen these data, we constructed expression plasmids expressing a catalytically inactive mutant of TRIM27 (C16A&C31A) and found that both a catalytically inactive mutation (C16A&C31A) and deletion of the E3 catalytic domain (ΔRING) led to loss of the suppressive effect of TRIM27 on IKKα (Figure 2E; Figure S5E). Consistent with the above findings, the overexpression of WT but not catalytically inactive TRIM27 (ΔRING, C16A&C31A) facilitated the K48‐linked polyubiquitination of IKKα, whereas TRIM27 knockdown downregulated the ubiquitination of IKKα (Figure 2F–H; Figure S5F). Next, we explored whether TRIM27 could affect the expression of non‐canonical NF‐κB molecules, and found that the expression of p100 and p52 were not significantly altered by TRIM27 (Figure S6A). The above results further demonstrate that TRIM27 has an important biological function on promoting IKKα protein degradation.

Next, we determined the specific sites at which IKKα is ubiquitinated by TRIM27. To this end, we generated a series of truncated forms of IKKα and examined the ubiquitination levels of these truncated IKKα proteins in HEK293T cells transfected with TRIM27. Interestingly, only the 500–746 truncation resulted in increased ubiquitination with the overexpression of TRIM27, whereas TRIM27 overexpression failed to promote the ubiquitination of the other two truncated IKKα proteins (1‐371 and 371–500) (Figure 2I). These results indicate that the lysine residues in the 500–746 region of TRIM27 could be potential ubiquitination sites for IKKα by TRIM27. To further determine the sites ubiquitinated by TRIM27, we constructed several IKKα mutants: K506R, K569R, K572R, K602R, K615R, K617R, and K624R. We found that TRIM27 failed to increase the K48‐linked polyubiquitination level of the IKKα‐K569R mutant (Figure 2J; Figure S5G). Additionally, CHX assays revealed that the half‐life of the IKKα‐K569R mutant protein was not affected by TRIM27 overexpression (Figure 2K). TRIM27 overexpression also promoted the protein degradation of WT IKKα but not the IKKα‐K569R mutant (Figure S5H). Taken together, these data indicate that TRIM27 can ubiquitinate IKKα at lysine residue 569 to promote the degradation of IKKα in IECs.

### 2.3 TRIM27 Promotes TRAF6 Degradation by Ubiquitination at Lysine Residue 489

**FIGURE 3:**
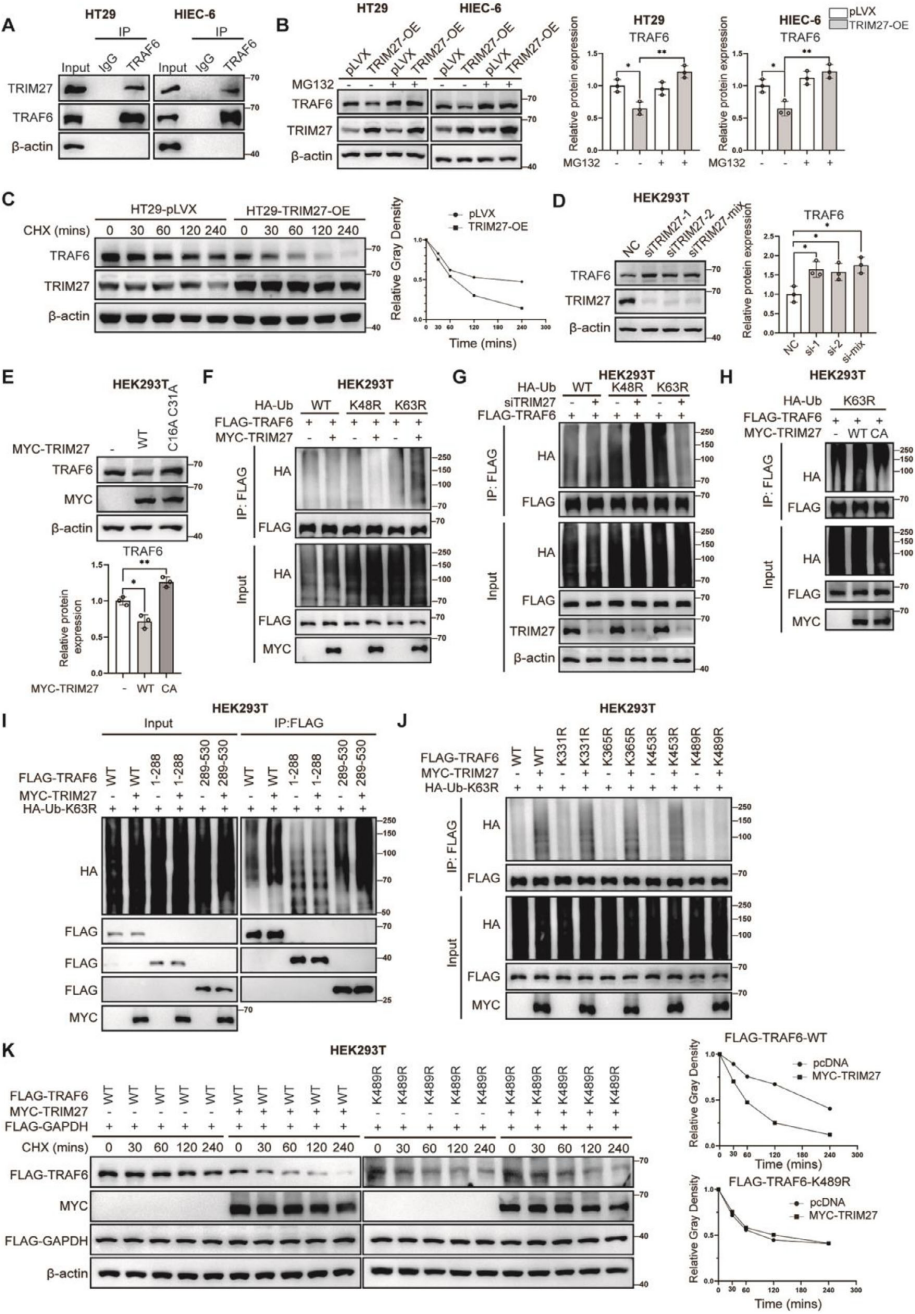
TRIM27 promotes TRAF6 degradation by ubiquitination at lysine residue 489. (A) Endogenous Co‐IP of TRIM27 and TRAF6 in HT29 and HIEC‐6 cells. (B) The protein levels of TRAF6 were detected in control and TRIM27‐overexpressing HT29 and HIEC‐6 cells with or without MG132 treatment by western blots. (C) Analysis of the TRAF6 protein half‐life in control and TRIM27‐overexpressing HT29 cells. Cells were treated with CHX for the indicated times before western blot analysis of IKKα expression. (D) The TRAF6 protein levels were examined in HEK293T cells transfected with control and siRNA targeting TRIM27 by western blots. (E) HEK293T cells were transfected with the WT and the E3 ligase dominant negative mutant (C16A& C31A) of MYC‐TRIM27. TRAF6 expression was examined by western blots. (F) HEK293T cells were transfected with HA‐Ub, HA‐Ub‐K48R, HA‐Ub‐K63R, FLAG‐TRAF6, and MYC‐TRIM27. Cell lysates were used for immunoprecipitation of FLAG‐TRAF6 and subsequent quantification of the ubiquitination level by western blots. (G) The control and TRIM27‐delepted HEK293T cells were transfected with HA‐Ub, HA‐Ub‐K48R, HA‐Ub‐K63R, and FLAG‐TRAF6. FLAG‐TRAF6 was immunoprecipitated for subsequent quantification of the ubiquitination level of TRAF6 by western blots. (H) HEK293T cells were transfected with HA‐Ub‐K63R, FLAG‐TRAF6, MYC‐TRIM27 and MYC‐TRIM27^C16A&C31A^. The ubiquitination level of TRAF6 was analyzed as described above. (I) HEK293T cells were transfected with HA‐Ub‐K63R, full‐length and 1–288, 289–530 truncation of FLAG‐TRAF6 with or without coexpression of MYC‐TRIM27. Cell lysates were used for immunoprecipitation of FLAG‐TRAF6 and subsequent quantification of the ubiquitination level via western blotting. (J) HEK293T cells were transfected with HA‐Ub‐K63R, FLAG‐TRAF6^WT^, FLAG‐TRAF6^K331R^, FLAG‐TRAF6^K365R^, FLAG‐TRAF6^K453R^, FLAG‐TRAF6^K489R^, and MYC‐TRIM27. Cell lysates were used for immunoprecipitation of FLAG‐TRAF6 and subsequent quantification of the ubiquitination level via western blotting. (K) Analysis of the half‐life of the WT and K489R mutant FLAG‐TRAF6 in HEK293T cells transfected with control and MYC‐TRIM27 plasmids. Cells were treated with CHX for the indicated times before western blot analysis. One‐way ANOVA (B, D, E) was performed to assess statistical significance. * *p* < 0.05, ** *p* < 0.01, *** *p* < 0.001.

TRAF6 is a RING‐finger‐dependent E3 ubiquitin ligase that is crucial for the activation of the IKK complex in the IL‐1R and TLR4 signaling pathways [14]. Since TRIM27 interacts with multiple components of the NF‐κB pathway, we screened in‐house plasmids related to the NF‐κB pathway and identified new interactions between TRIM27 and TRAF6 by exogenous, semiendogenous co‐IP in HEK293T cells (Figure S7A,B). The interactions between endogenous TRIM27 and TRAF6 were confirmed in HT29 and HIEC‐6 cells (Figure 3A). Similar to the interaction between TRIM27 and IKKα, both the N‐terminal RING‐finger (1‐62) and coiled coil (133‐295) domains of TRIM27 are involved in the interaction with TRAF6 (Figure S7C).

Next, we determined the effects of TRIM27 on the protein stability and ubiquitination of TRAF6. As shown in Figure 3B, forced expression of TRIM27 mildly decreased the protein level of TRAF6, which was abrogated by MG132 treatment. CHX assays further revealed that TRIM27 overexpression resulted in a decreased half‐life of endogenous TRAF6 in HT29 and HIEC‐6 cells (Figure 3C; Figure S7D). As expected, TRIM27 knockdown increased the protein level of TRAF6, and the catalytically inactive TRIM27^C16A&C31A^ and TRIM27^ΔRING^ did not affect the TRAF6 protein level (Figure 3D,E; Figure S7E). Intriguingly, the total ubiquitination level of TRAF6 was not significantly altered by TRIM27 overexpression, whereas the non‐K63‐linked polyubiquitination level was markedly increased, but the non‐K48‐linked polyubiquitination level was decreased by TRIM27 overexpression (Figure 3F). In contrast, TRIM27 knockdown led to downregulated non‐K63‐linked polyubiquitination and facilitated non‐K48‐linked polyubiquitination (Figure 3G). Since the decrease in TRAF6 protein expression induced by TRIM27 overexpression was impaired by MG132 treatment, we hypothesized that the enhanced non‐K63‐linked polyubiquitination of TRAF6 by TRIM27 was at least part of the K48‐linked polyubiquitination, which resulted in proteosome‐dependent degradation of TRAF6. Consistent with these findings, overexpression of the catalytically inactive TRIM27 failed to regulate the non‐K63‐linked ubiquitination level of TRAF6 (Figure 3H; Figure S7F).

We also identified the specific sites at which TRAF6 is ubiquitinated by TRIM27. As shown in Figure 3I, the non‐K63‐linked polyubiquitination of the 289–530 truncation of TRAF6 was still significantly increased after transfection with TRIM27. To further determine the specific lysine residue in the 289–530 region of TRAF6 that is ubiquitinated by TRIM27, we constructed a series of TRAF6 mutants: K331R, K365R, K453R and K489R. We found that TRIM27 failed to promote the non‐K63‐linked polyubiquitination of TRIM27^K489R^ (Figure 3J). Additionally, TRIM27 overexpression did not affect the half‐life or the protein level of the TRAF6^K489R^ mutant (Figure 3K; Figure S7G). Taken together, these results indicate that TRAF6 is a substrate of TRIM27, which could also be involved in modulating the NF‐κB pathway in IECs.

### 2.4 TNF‐α Destabilizes the TRIM27 Protein in IECs

**FIGURE 4:**
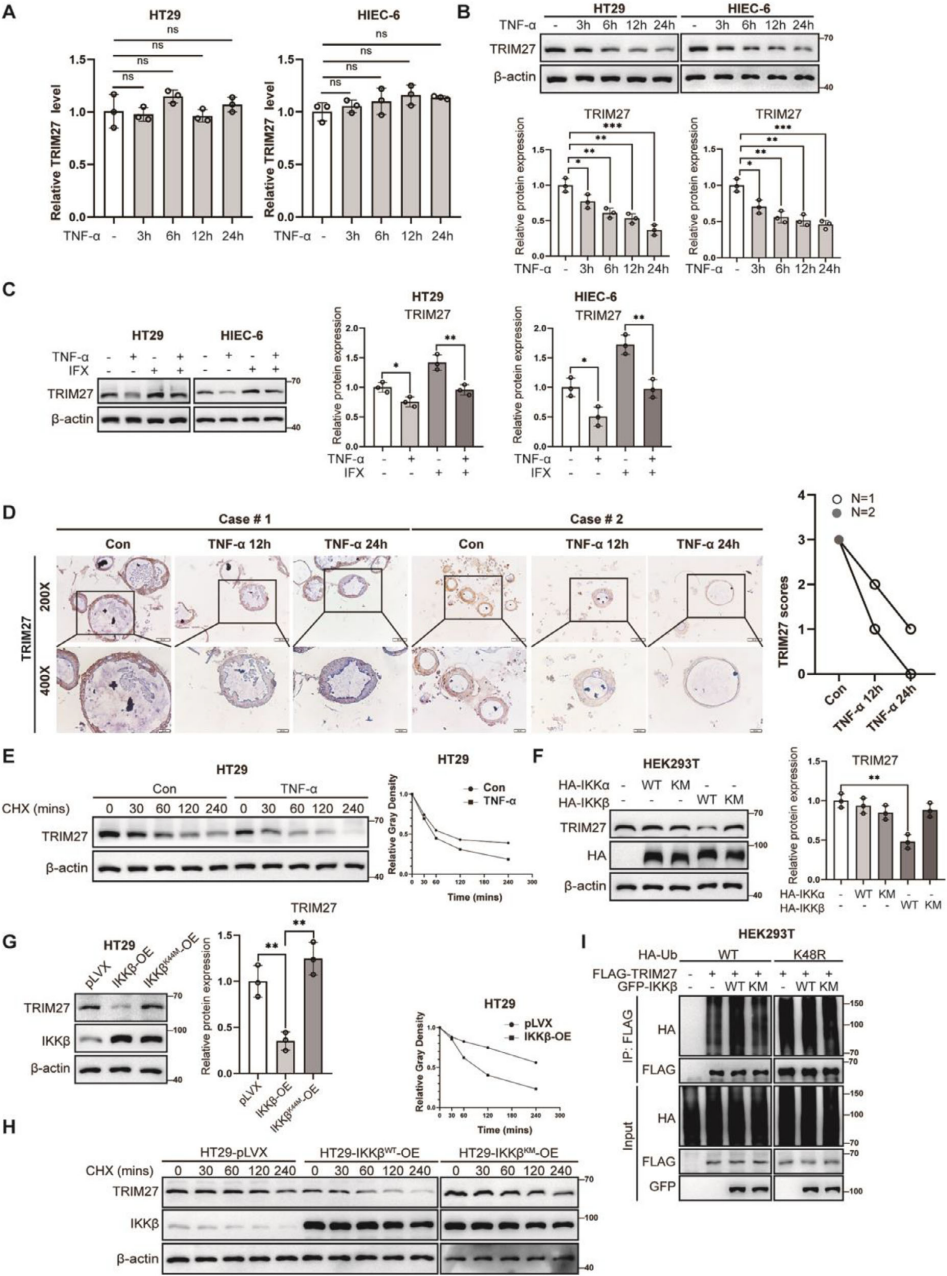
TNF‐α destabilizes the TRIM27 protein in IECs. (A) The mRNA level of TRIM27 in HT29 and HIEC‐6 cells stimulated with TNF‐α for the indicated times was analyzed by qRT‒PCR. (B) Western blot analysis of TRIM27 expression in HT29 and HIEC‐6 cells treated with TNF‐α (10 ng mL^−1^) for the indicated times. (C) Western blot analysis of TRIM27 expression in HT29 and HIEC‐6 cells treated with TNF‐α (10 ng mL^−1^) alone or in combination with IFX (10 µg mL^−1^) for the indicated times. (D) TRIM27 expression was examined in human colonoids after exposure to TNF‐α (10 ng mL^−1^) for the indicated times by IHC. (E) Analysis of the half‐life of the TRIM27 protein in HT29 cells with or without TNF‐α (10 ng mL^−1^) treatment. The cells were treated with CHX (75 µg mL^−1^) for the indicated times before western blot analysis of TRIM27 expression. (F) Western blot analysis of TRIM27 expression in HEK293T cells transfected with the control vector, the HA‐IKKα/β WT plasmid or the kinase‐dead mutant (K44M) plasmid. (G) Western blot analysis of TRIM27 expression in HT29 cells stably overexpressing IKKβ^WT^ or IKKβ^K44M^ and the corresponding control cells. (H) Analysis of the TRIM27 protein half‐life in control and IKKβ^WT^ or IKKβ^K44M^‐overexpressing HT29 cells. The cells were treated with cycloheximide (CHX) (75 µg mL^−1^) for the indicated times before western blot analysis of TRIM27 expression. (I) HEK293T cells were transfected with HA‐Ub, HA‐Ub‐K48R, FLAG‐TRIM27, MYC‐IKKβ^WT^, and MYC‐IKKβ^K44M^. The cell lysates were used for immunoprecipitation of FLAG‐TRIM27 and subsequent quantification of the ubiquitination level by western blotting. The data are presented as the means ± SDs and were from three independent experiments. One‐way ANOVA (A‐C, F, G) was performed to assess statistical significance.

Next, we investigated the expression of TRIM27 in IECs during intestinal inflammation. Interestingly, we found that the mRNA level of TRIM27 was not significantly altered by TNF‐α treatment (Figure 4A), whereas TNF‐α‐induced downregulation of TRIM27 protein levels was largely abrogated by infliximab (IFX) treatment in HT29 and HIEC‐6 cells (Figure 4B,C). These data suggest that an alteration in protein stability could account for the downregulation of TRIM27 during intestinal inflammation. IHC analysis of human colonoids also revealed the downregulation of TRIM27 after exposure to TNF‐α (Figure 4D). Not surprisingly, in response to TNF‐α treatment, the half‐life of endogenous TRIM27 was markedly decreased (Figure 4E; Figure S8A). Because TRIM27 interacts with the IKK complex and TRIM27 was indicated to be a substrate of IKKs in an in vitro kinase array [11], we hypothesized that the IKK complex may phosphorylate and destabilize TRIM27. To this end, we explored the effect of IKK overexpression on endogenous TRIM27 in HEK293T cells. Interestingly, only WT‐IKKβ, but not IKKα or kinase‐dead IKKβ (K44M), significantly decreased the protein level of TRIM27 (Figure 4F). Similar results were observed in HT29 and HIEC‐6 cells stably overexpressing IKKβ^WT^ or IKKβ^K44M^ (Figure 4G; Figure S8B). Ubiquitination and CHX assays further demonstrated that IKKβ^WT^ but not its kinase‐dead mutant IKKβ^K44M^ promoted K48‐linked ubiquitination of TRIM27 and significantly decreased the half‐life of endogenous TRIM27 in HT29 and HIEC‐6 cells (Figure 4H,I; Figure S8C). Co‐IP further confirmed the interaction between IKKβ and TRIM27 and demonstrated that TRIM27 shared the interfaces to interact with both IKKα and IKKβ, through overexpression of IKKα didn't modulate TRIM27 protein level (Figure S8D–G). Taken together, these data suggest that TRIM27 in IECs is inhibited during intestinal inflammation, likely through the activation of IKKβ kinase.

### 2.5 IKKβ Phosphorylates and Destabilizes TRIM27 by Disrupting the USP7‐TRIM27 Complex and Antagonizing USP7‐Mediated Deubiquitination

**FIGURE 5:**
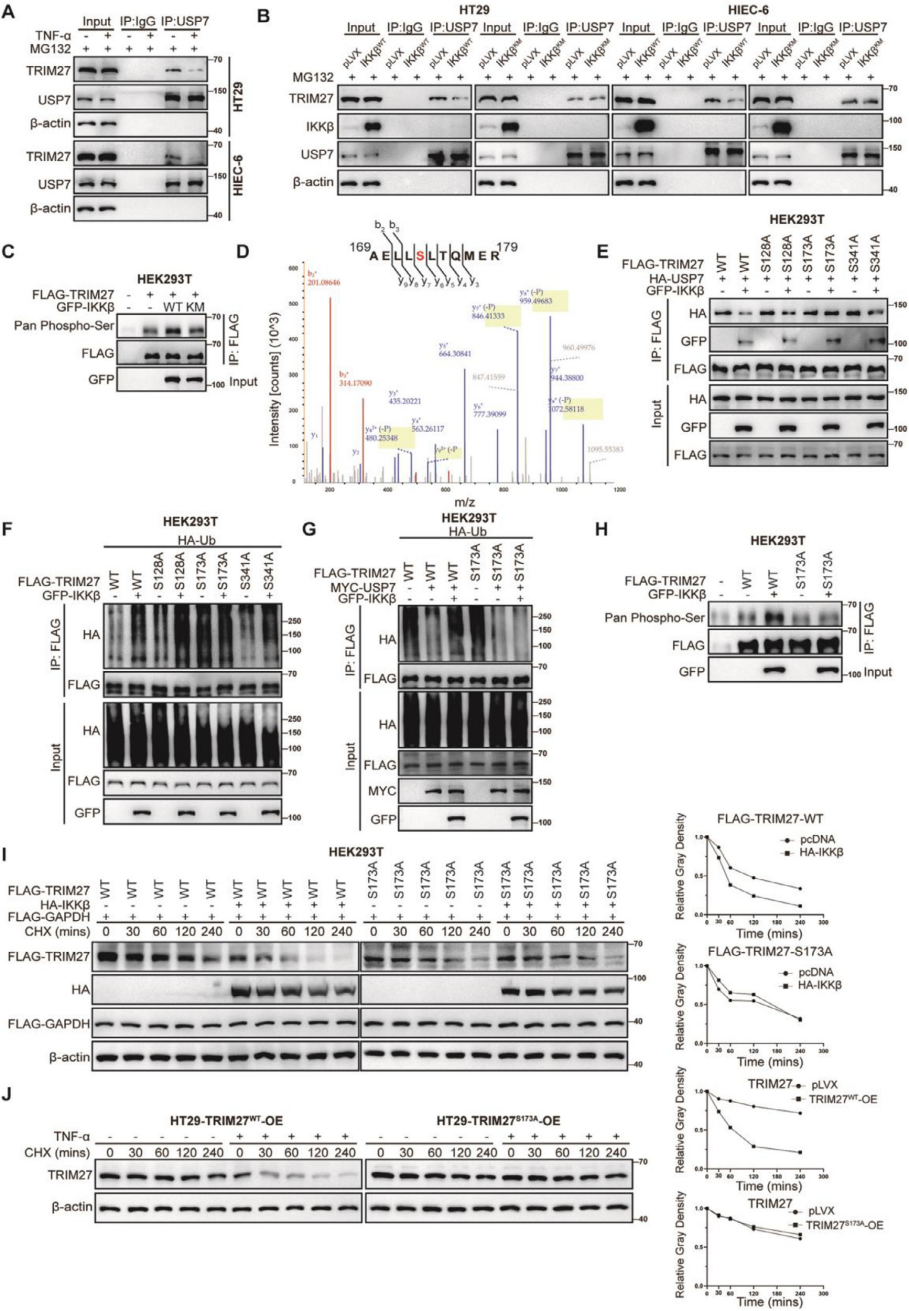
IKKβ phosphorylates and destabilizes TRIM27 via disrupting USP7‐TRIM27 complex and antagonizing USP7‐mediated deubiquitination. (A) Endogenous co‐IP of TRIM27 and USP7 in HT29 and HIEC‐6 cells with or without TNF‐α (10 ng mL^−1^) treatment for 24 h. The cells were treated with 10 µM MG132 for 6 h before lysis for subsequent analysis. (B) Endogenous co‐IP of TRIM27 and USP7 in HT29 and HIEC‐6 cells with and without IKKβ^WT^ or IKKβ^K44M^ overexpression. The cells were treated with 10 µM MG132 for 6 h before lysis for subsequent analysis. (C) HEK293T cells were transfected with FLAG‐TRIM27, GFP‐IKKβ^WT^ or GFP‐IKKβ^K44M^ plasmids. Cell lysates were used for immunoprecipitation with an anti‐FLAG antibody to detect the level of pan‐phosphorylated serine. (D) Identification of phosphorylated TRIM27‐S173 in control and IKKβ‐overexpressing HEK293T cells with transfection of FLAG‐TRIM27 by tandem mass spectrometry. (E) Exogenous co‐IP of HA‐USP7 and WT, S128A, S173A, and S341A mutant of FLAG‐TRIM27 with or without transfection of GFP‐IKKβ. (F) HEK293T cells were transfected with HA‐Ub, GFP‐IKKβ, FLAG‐TRIM27^WT^, FLAG‐TRIM27^S128A^, FLAG‐TRIM27^S173A^, and FLAG‐TRIM27^S341A^. FLAG‐TRIM27 was immunoprecipitated for subsequent quantification of the ubiquitination level of TRIM27 by western blotting. (G) HEK293T cells were transfected with the indicated HA‐Ub, MYC‐USP7, GFP‐IKKβ, WT, and S173A mutant of FLAG‐TRIM27 plasmids. Cell lysates were used for immunoprecipitation of FLAG‐TRIM27 and subsequent quantification of the ubiquitination level by western blotting. (H) HEK293T cells were transfected with GFP‐IKKβ, WT and S173A mutant of FLAG‐TRIM27. Cell lysates were used for immunoprecipitation with an anti‐FLAG antibody to detect the level of pan‐phosphorylated serine. (I) Analysis of the half‐life of the WT and S173A mutant FLAG‐TRIM27 in HEK293T cells transfected with control and HA‐IKKβ plasmids. Cells were treated with CHX (75 µg mL^−1^) for the indicated times before western blot analysis. (J) Analysis of the TRIM27 protein half‐life in control and TRIM27^WT^ or TRIM27^S173A^‐overexpressing HT29 cells with or without TNF‐α (10 ng mL^−1^) treatment. Cells were treated with CHX (75 µg mL^−1^) for the indicated times before western blot analysis of TRIM27 expression.

Previous studies have revealed that ubiquitin‐specific protease 7 (USP7) is a deubiquitinating enzyme for TRIM27 that prevents TRIM27 autoubiquitination and protein degradation [15, 16, 17]. Thus, we explored whether IKKβ modulates the formation of the USP7/TRIM27 complex to destabilize the TRIM27 protein. We observed that the binding of USP7 to TRIM27 was significantly reduced after TNF‐α stimulation in HT29 and HIEC‐6 cells (Figure 5A). Exogenous and semiendogenous co‐IP assays in HEK293T cells further demonstrated that the suppressive effect of IKKβ on the formation of the USP7‐TRIM27 complex required its kinase activity (Figure S9A). Similar results were observed in HT29 and HIEC‐6 cells overexpressing IKKβ^WT^ or IKKβ^K44M^ (Figure 5B). Consistently, overexpression of USP7 led to decreased ubiquitination and increased protein expression of TRIM27, which was abolished by co‐expression of IKKβ^WT^ but not IKKβ^K44M^ (Figure S9B,C). In contrast, treatment with the USP7 inhibitor P5091 or USP7 knockdown resulted in decreased TRIM27 protein levels; however, overexpression of IKKβ or TNF‐α did not affect TRIM27 protein levels in cells with P5091 or USP7 knockdown (Figure S9D–G). Indeed, P5091 or USP7 knockdown increased the level of ubiquitinated TRIM27, which was not affected by IKKβ (Figure S9H,I). These results indicated that IKKβ‐induced downregulation of TRIM27 protein could be dependent on USP7.

In line with previous reports of TRIM27 phosphorylation by IKKβ in vitro [11], we observed that overexpression of IKKβ^WT,^ but not IKKβ^K44M^, increased the level of TRIM27 phosphorylation by a panphospho‐serine antibody (Figure 5C). To identify the specific site of TRIM27 phosphorylated by IKKβ, we performed IP‒MS/MS analysis of TRIM27 in HT29 cells stably expressing FLAG‐TRIM27 after TNF‐α stimulation and found that three sites (S128, S173 and S341) could be candidates for TRIM27 phosphorylation by IKKβ (Figure 5D; Figure S10A and Table S1).

Interestingly, we observed that only the S173A but not the S128A and S341A mutants abolished the inhibitory effects of IKKβ on the interaction between TRIM27 and USP7, as well as USP7‐mediated TRIM27 deubiquitination (Figure 5E,F). Similarly, overexpression of USP7 decreased the ubiquitination level of both WT and S173A mutant TRIM27, whereas only the USP7‐induced deubiquitination of WT TRIM27 was largely abrogated by IKKβ overexpression (Figure 5G). Consistent with the above results, the S173A mutation abolished the increase in TRIM27 phosphorylation caused by IKKβ overexpression (Figure 5H). Furthermore, the phosphomimetic S173E mutant of TRIM27 largely lost its ability to interact with USP7 and USP7‐mediated deubiquitination (Figure S10B,C). In addition, IKKβ or TNF‐α stimulation failed to further decrease the half‐life of TRIM27 or the TRIM27 protein level in IECs when S173‐TRIM27 was mutated (Figure 5I,J; Figure S10D,E). Taken together, these results indicate that S173‐TRIM27 phosphorylation by IKKβ kinase decreases its binding to USP7, which attenuates USP7‐mediated TRIM27 deubiquitination and subsequently promotes TRIM27 degradation.

### 2.6 TRIM27 Enhances the Therapeutic Effect of IFX on Intestinal Inflammation in IECs

**FIGURE 6:**
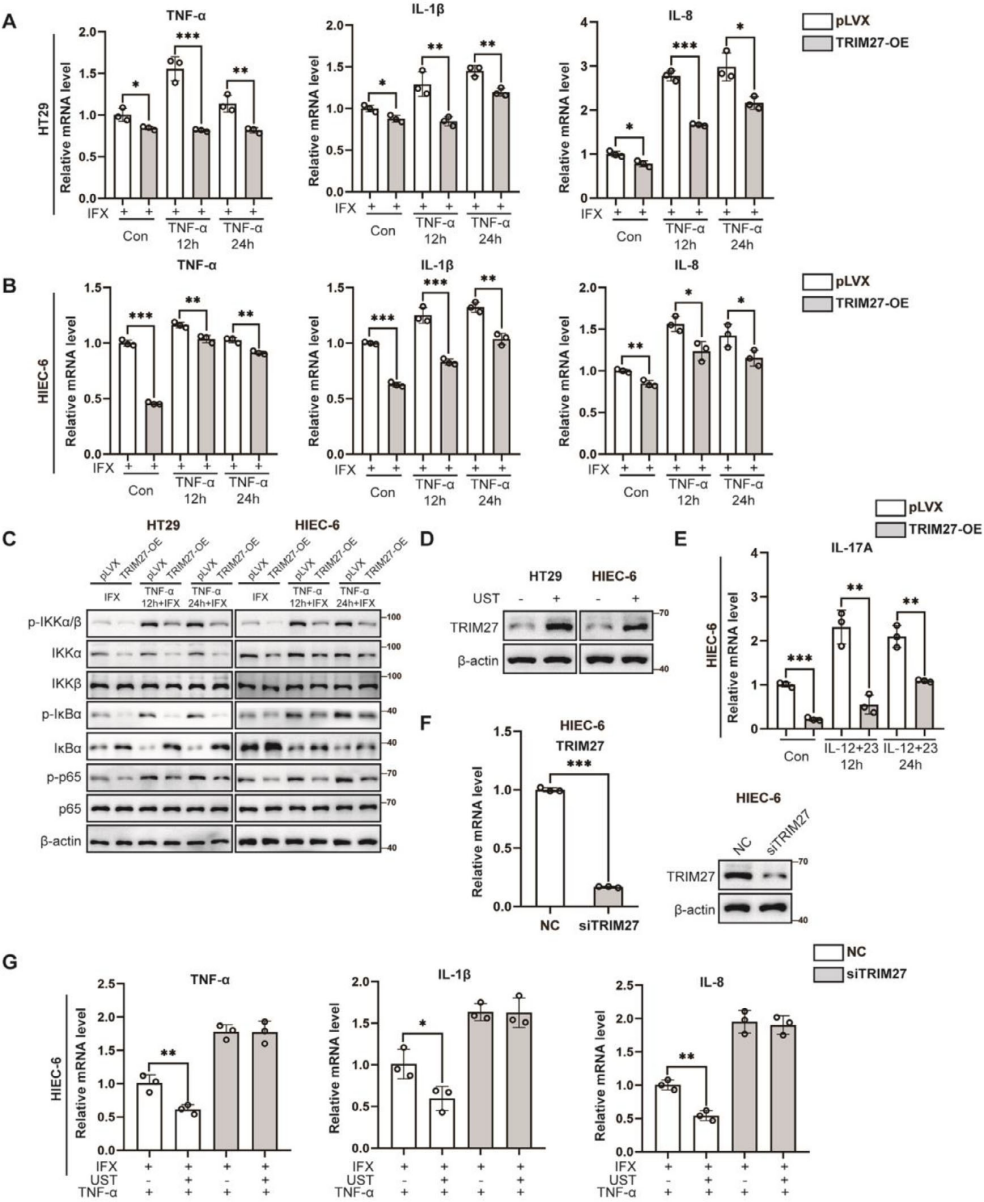
TRIM27 enhances the therapeutic effect of IFX on intestinal inflammation in IECs. (A,B) The mRNA levels of proinflammatory cytokines were measured by qRT‒PCR in control and TRIM27‐overexpressing HT29 and HIEC‐6 cells after treatment with TNF‐α (10 ng mL^−1^) and IFX (10 µg mL^−1^) for the indicated times. (C) Immunoblotting was performed to measure the levels of p‐IKKα/β, IKKα, IKKβ, p‐IκBα, IκBα, p‐p65 and p65 in control and TRIM27‐overexpressing HT29 and HIEC‐6 cells after TNFα stimulation and treatment with IFX (10 µg mL^−1^) for the indicated times. (D) Western blot analysis of TRIM27 expression in HT29 and HIEC‐6 cells treated with UST (90 µg mL^−1^) for 24 h. (E) The mRNA levels of IL‐17A were measured by qRT‒PCR in TRIM27‐overexpressing and control HIEC‐6 cells after exposure to IL‐12 (10 ng mL^−1^) and IL‐23 (10 ng mL^−1^) for the indicated times. (F) The efficiency of TRIM27 knockdown by transfection of siRNA targeting TRIM27 was examined in HIEC‐6 cells by qRT‒PCR and western blot analysis. (G) The mRNA levels of proinflammatory cytokines were measured by qRT‒PCR in control and TRIM27‐knockdown HIEC‐6 cells after treatment with TNF‐α (10 ng/ml) and IFX (10 µg mL^−1^) alone or in combination with UST (90 µg mL^−1^) for 24 h. The data are presented as the means ± SDs. *n* = 3 (A, B, E–G) biologically independent samples per group. The data (A, B, E–G) are representative of 3 independent experiments. One‐way ANOVA (A, B, E, G) and two‐tailed, unpaired Student's t test (F) were performed to assess statistical significance. * *p* < 0.05, ** *p* < 0.01, *** *p* < 0.001.

Since our findings demonstrated the suppressive effect of epithelial TRIM27 on intestinal inflammation, the expression of which is downregulated in response to TNF‐α, we aimed to determine whether re‐expression of TRIM27 in IECs could enhance the therapeutic effect of IFX. To test this hypothesis, HT29 cells, HIEC‐6 cells with forced expression of TRIM27 and control cells were treated with TNF‐α and IFX or IFX alone. We found that TNF‐α led to mildly upregulated transcription of proinflammatory cytokine genes and phosphorylation of IKKα/β, IκBα, and p65 in IECs treated with IFX, indicating basal activation of NF‐κB signaling or activation of IKKs by alternative signaling (Figure 6A–C). However, TRIM27 overexpression further downregulated the mRNA levels of proinflammatory cytokine genes and the phosphorylation levels of IKKα/β, IκBα, and p65 in IECs subjected to IFX treatment (Figure 6A–C). These data suggest that increased expression of TRIM27 could synergize with IFX to inhibit intestinal inflammation.

Ustekinumab (UST) is a monoclonal antibody that targets the p40 subunit of IL‐12 and IL‐23 and has shown promising efficacy in both CD and moderate‐to‐severe UC [18, 19]. By serendipity, we found that UST treatment markedly induced TRIM27 expression in HT29 and HIEC‐6 cells, indicating that IECs could be potential target cells for UST (Figure 6D). IL‐12/23 treatment induced the transcriptional activation of proinflammatory cytokine genes downstream of the IL‐12/23 pathway, such as IL‐17A, in HIEC‐6 cells, indicating that IECs can also respond to IL‐12/23 stimulation (Figure 6E). Importantly, TRIM27 overexpression considerably inhibited the transcriptional activation of the proinflammatory cytokine IL‐17A in HIEC‐6 cells (Figure 6E). On the basis of these findings, we hypothesized that the therapeutic effect of combined IFX and UST treatment may be dependent on UST‐induced TRIM27 expression. Indeed, knockdown of TRIM27 impaired the enhanced inhibitory effect of UST plus IFX on the TNF‐α‐induced inflammatory response in HIEC‐6 cells (Figure 6F,G). Thus, these data suggest that the upregulation of TRIM27, such as via coadministration with UST, could improve the efficacy of IFX in inhibiting intestinal inflammation. The bar graphs showing grayscale value of WB data of Figure 6 were shown in Supporting files.

### 2.7 TRIM27 Expression is Decreased in UC

**FIGURE 7:**
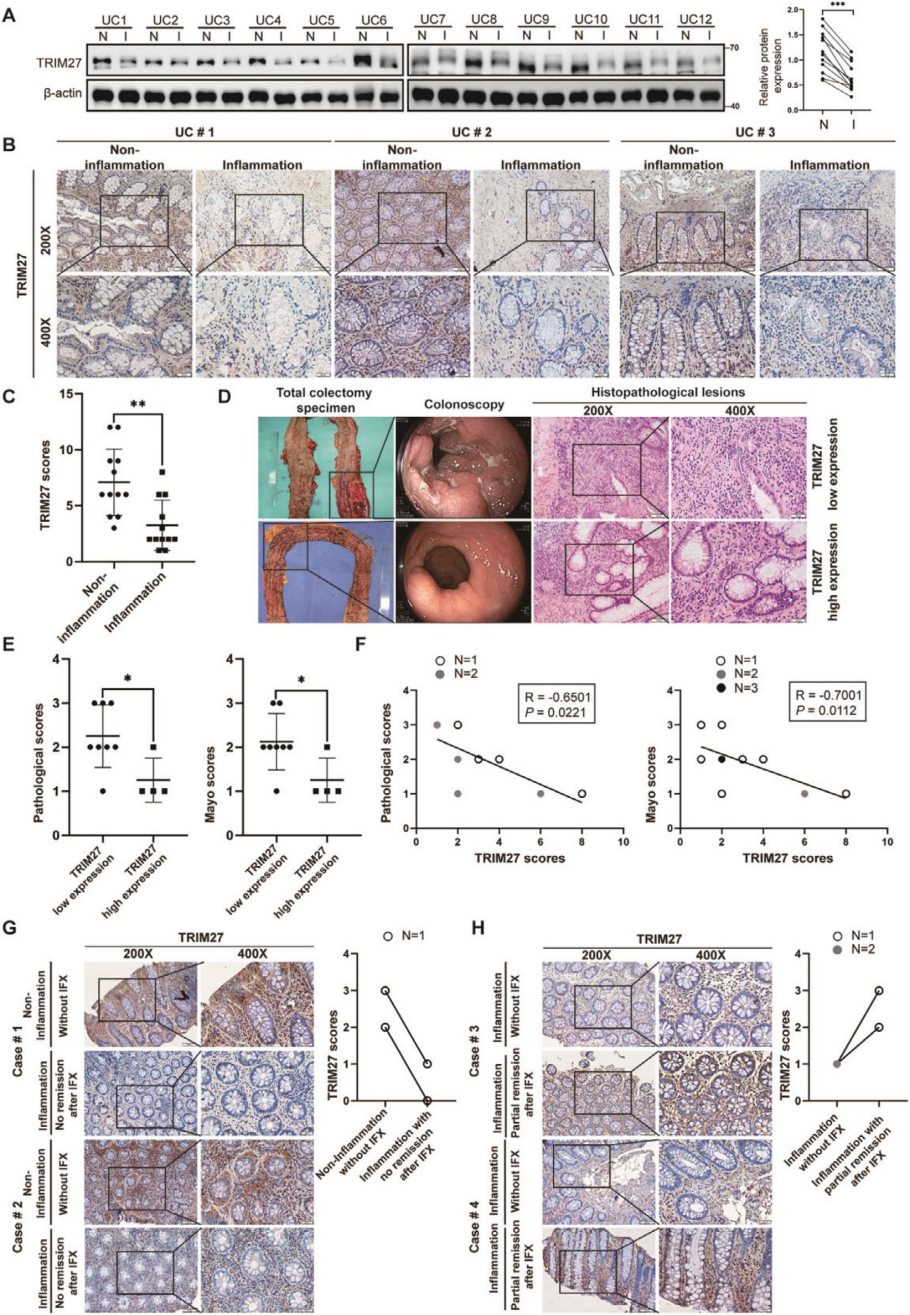
TRIM27 expression is decreased in inflamed colon tissues from UC patients. (A) TRIM27 expression was examined in inflamed and paired adjacent noninflamed colon tissues from patients with UC by western blot analysis (*n* = 12). (B,C) TRIM27 expression was examined, evaluated and semiquantitatively scored in inflamed and paired adjacent noninflamed colon tissues from patients with UC by IHC (*n* = 12). (D) Representative gross colorectal specimen, endoscopic severity and hematoxylin–eosin staining images of inflamed colon tissues with low and high TRIM27 expression. (E) Histological pathological scores and endoscopic severity assessed by the Mayo endoscopic score (MES) were assessed in inflamed colon tissues with low and high TRIM27 expression. (F) Spearman correlation analysis was used to evaluate the correlations between TRIM27 immunohistochemical scores and pathological scores and the MES in inflamed colon tissues from patients with UC (*n* = 12). (G) TRIM27 expression was examined in noninflamed and inflamed colon tissues from patients with UC without clinical remission after IFX treatment. (H) TRIM27 expression was examined in inflamed colon tissues and colon tissues from patients with UC partial clinical remission after IFX treatment. The data are presented as the means ± SDs. Two‐tailed, unpaired Student's t test (A), the Wilcoxon signed‐rank test (C) and Mann–Whitney U test (E) were performed to assess statistical significance. *p < 0.05, **p < 0.01.

To explore whether TRIM27 expression is dysregulated in UC, we first examined the TRIM27 protein level in inflamed and noninflamed intestinal tissues from UC patients. Both western blot and immunohistochemical analyses revealed that the protein levels of TRIM27 were moderately decreased in the inflamed intestines (Figure 7A–C). Moreover, the histological pathological score and endoscopic severity assessed by the Mayo endoscopic score (MES) were lower in inflamed colon tissues with high TRIM27 expression, which was significantly negatively correlated with the IHC score of TRIM27 (R = ‐0.6501, p = 0.0221 for histological pathological scores; R = ‐0.7001, p = 0.0112 for the MES) (Figure 7D–F). Next, we assessed TRIM27 expression in UC patients treated with IFX. In the intestinal tissues from two patients who did not achieve clinical remission after IFX treatment, TRIM27 expression was still low in the inflamed intestines (Figure 7G). Notably, TRIM27 expression in inflamed intestines was moderately increased in two patients who achieved clinical partial remission after IFX treatment (Figure 7H). Taken together, these results indicate that decreased expression of TRIM27 may be associated with aggravated endoscopic severity, histopathological lesions, and impaired therapeutic effects of IFX in UC, which further supports enhancing TRIM27 expression as a potential strategy for refractory patients with UC.

## 3 Discussion

As a member of the E3‐ligase TRIM family, TRIM27 is a multifunctional protein that has long been known as a negative modulator of the NF‐κB pathway and the innate immune response [11, 20]. In CD4 T cells, TRIM27 mediates lysine 48 polyubiquitination and promotes protein degradation of PI3KC2β, which further inhibits KCa3.1 channel activity and T cells’ activation [21]. However, in contrast to its role as a negative regulator of the proinflammatory NF‐κB pathway and CD4 T cells, Trim27^−/−^ mice are not susceptible to DSS‐induced colitis, which is due to decreased STAT3 activation by TRIM27 in hematopoietic cells and suggests a proinflammatory function of TRIM27 in immune cells during the development of IBD [10]. Notably, STAT3 signaling plays dual roles in colitis [22, 23, 24]. Epithelial STAT3 promotes epithelial survival, proliferation and repair, and mice with epithelial knockout of STAT3 are susceptible to colitis [23, 24]. Indeed, our study revealed the anti‐inflammatory function of TRIM27 in IECs. Although NF‐κB is also a key modulator of intestinal epithelial survival and apoptosis, both overactivation and defects in the epithelial NF‐κB predispose individuals to intestinal colitis [25, 26]. Thus, epithelial knockout of *Trim27* enhances NF‐κB activity in IECs and promotes intestinal inflammation. The increased expression of proinflammatory cytokines by activated NF‐κB in *Trim27* KO IECs could result in the impairment of tight junctions and intestinal barriers and the presence of ulcer‐type lesions in UC. Notably, although TRIM27 is increased in MLN CD4+ T lymphocytes in a DSS‐induced colitis model, the upregulation of TRIM27 expression could constitute negative feedback and compensate for the excess activation of CD4+ T cells in IBD [12]. In contrast to previous reports that TRIM27 is overexpressed in CD, our finding that TRIM27 is downregulated in UC is in line with the anti‐inflammatory function of epithelial TRIM27 in colitis.

In the cytoplasm, TRIM27 interacts with both IKK kinases and TBK1, which leads to the inhibition of NF‐κB‐ and IRF3‐dependent proinflammatory transcriptional activation [11]. Although ISRE reporter assays have revealed the RING finger‐independent mechanism of TRIM27‐mediated inhibition of IKK‐mediated ISRE activation, the mechanism underlying the negative regulation of NF‐κB is largely unknown [11]. The pioneering study reported that TRIM27 did not affect the expression of IKKs, which was dependent on experiments involving transient co‐overexpression of both TRIM27 and individual IKK kinases in HEK293T cells [11]. However, the excess endogenous protein levels of overexpressed IKKs could mask the effect of TRIM27 on the protein degradation of IKKs. Here, we found that TRIM27 functions as an E3 ligase of IKKα and TRAF6 to inhibit NF‐κB activation in IECs. Intriguingly, the ubiquitination of IKKβ is not regulated by TRIM27, although TRIM27 also interacts with IKKβ. One possibility is that K569 (^566^DLYKQLK^572^), the ubiquitination site of IKKα, is not conserved in IKKβ, in which arginine 572 (^569^ELYRKLR^575^) replaces the corresponding lysine in IKKα. Future structural and biochemical studies exploring the TRIM27‐IKKα/β complex could shed light on the mechanism underlying the discrepant regulation of IKKα/β by TRIM27.

As a multifunctional and vital protein in the immune response, TRIM27 is also dynamically regulated [20]. As discussed above, the upregulation of TRIM27 is thought to be negative feedback to underlying the excess activation of CD4 T cells in IBD. Similarly, a comparison of the transcriptional profiles of CD4 T cells derived from the lamina propria and epithelium revealed that TRIM27 is transcriptionally upregulated in CD4 T cells during lamina propria‐to‐epithelium translocation, supporting a negative feedback loop in the lamina propria‐to‐epithelium translocation [27]. Additionally, type I IFN induces the downregulation of miRNA‐27a, which targets and suppresses TRIM27 expression [28]. Thus, type I IFN upregulates TRIM27, a negative regulator of type I IFN production, resulting in negative feedback [28]. In addition to the transcriptional and epigenetic regulation of TRIM27, the TRIM27 protein is protected from autoubiquitination and degradation by its binding partner USP7 [15, 17, 29]. Recently, WNK1 was found to facilitate the TRIM27/USP7 interaction and stabilize the TRIM27 protein in a kinase‐independent manner [15]. Since the mRNA level of TRIM27 is not altered during intestinal inflammation, in this study, we identified a kinase‐dependent posttranslational modification mechanism by which IKKβ disrupts the TRIM27/USP7 complex and destabilizes TRIM27 in response to TNF‐α, further indicating the vital role of USP7 in regulating TRIM27 expression. Moreover, our findings that TNFα induces TRIM27 degradation by IKKβ‐dependent phosphorylation of TRIM27 in IECs suggest a double‐negative feedback loop of the TRIM27‐IKK regulatory axis in the NF‐κB pathway, which could account for the downregulation of TRIM27 in UC. Notably, IKKβ but not IKKα downregulates the TRIM27 protein, which is consistent with the distinct phenotypes of IKKα or IKKβ gene knockout mice and the different requirements of IKKα and IKKβ in the canonical and noncanonical NF‐κB pathways in response to different stimuli [30, 31, 32].

TNF‐α is a critical cytokine involved in the initiation and development of UC [33, 34]. Although the advent of anti‐TNFα therapy via IFX was a major breakthrough for UC treatment, a significant proportion of patients still do not respond to IFX due to genetic factors, insufficient IFX serum levels or the development of antibodies to IFX, which leads to insufficient TNF‐α blockade and variable levels of sustained intestinal inflammation after anti‐TNFα therapy [35, 36, 37]. In this study, we observed that TRIM27 expression is notably decreased in inflamed intestines and that sustained downregulation of TRIM27 is associated with the limited therapeutic effect of IFX, whereas clinical remission after IFX treatment reactivates TRIM27 expression. Since TRIM27 is a negative regulator of IKKα and TRAF6, we hypothesize that decreased expression of TRIM27 may upregulate IKKα and TRAF6, which renders cells more sensitive to TNF‐α‐ and LPS‐induced NF‐κB activation and insensitive to anti‐TNFα therapy. Thus, re‐expression of TRIM27 is a potential strategy to enhance the therapeutic effect of IFX.

The cytokine IL‐23, which is predominantly produced by macrophages and dendritic cells, has emerged as a critical driver of chronic intestinal inflammation [38, 39]. IL‐23R inhibitors, such as ustekinumab and guselkumab, show promising efficacy in treating IBD, including refractory IBD resistant to TNF antagonists [18, 40, 41, 42, 43]. IL‐23 functions through the IL‐12Rβ1/IL‐23R heterodimeric receptor complex and is highly expressed in T cells, innate lymphoid cells, and relatively low levels in various immune cells and in IECs [38, 44]. Unexpectedly, we found that UST treatment dramatically induced TRIM27 expression in IECs, suggesting a strong biological effect of UST on IECs. Thus, UST treatment could be an effective way to re‐express TRIM27, and the upregulation of TRIM27 is a potential mechanism for UST to treat refractory IBD or enhance the therapeutic effect of IFX. Our current study revealed that the induced expression of TRIM27 is indispensable for the synergistic inhibitory effect of IFX and UST on proinflammatory gene expression in IECs. Currently, dual targeted therapy combining IFX and UST is considered a promising approach to improve the treatment outcomes of IBD patients. The efficacy and safety of IFX and UST combination therapy have been retrospectively confirmed in two cohorts [45, 46], and a prospective randomized trial has been initiated to evaluate the efficacy and safety of UST, IFX, and combination therapy [47]. Future studies regarding the molecular mechanism by which TRIM27 is regulated by UST and whether UST induces TRIM27 expression in immune cells are anticipated to provide new mechanistic insight into UST/IFX therapy and shed light on biomarker identification for the clinical assessment of UST/IFX therapy.

This study also has certain limitations. The fact that TRIM27 was not identified among the most significant sequencing results may affect the rationale for conducting this study. This discrepancy in intestinal epithelial inflammation between systemic TRIM27 knockout and intestinal epithelial‐specific TRIM27 knockout suggests that systemic knockout of TRIM27 may induce functional changes in cell types other than intestinal epithelial cells. Based on the pathological characteristics of intestinal epithelial inflammation and mucosal barrier damage in ulcerative colitis, we focused on studying molecules that function in the intestinal epithelium. The same molecule may exert distinct biological functions in different disease context and cell types. Therefore, based on the discrepancy between our preliminary experimental results and findings reported in the published literature, we initiated the current study to detect the function of TRIM27 on intestinal epithelial inflammation, which may explain the rationale behind undertaking this research. The fact that we did not identify TRIM27 among the most significantly differentially expressed molecules in the sequencing results is a limitation of this study. Molecules that are not the most significantly differentially expressed are not necessarily lacking important biological functions. In this study, we at least reveal the significant anti‐inflammatory function and mechanism of TRIM27 in the intestinal epithelium, as well as its clinical relevance to ulcerative colitis. These findings provide an experimental basis for understanding the pathogenesis and targeted therapy of ulcerative colitis.

**FIGURE 8:**
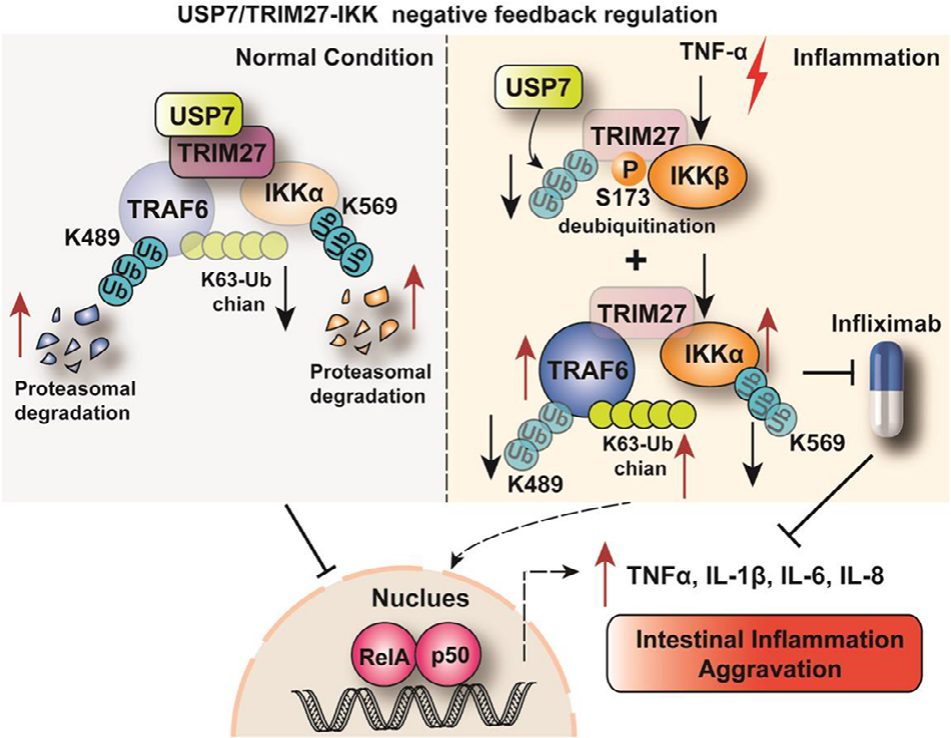
Schematic diagram of the findings in this study. A schematic model showing that TRIM27 inhibited intestinal inflammation and enhanced the therapeutic effect of infliximab by promoting the degradation of IKKα and TRAF6 by ubiquitinating at lysine residues 569 of IKKα and lysine residues 489 of TRAF6. Upon TNF‐α stimulation, activated IKKβ phosphorylates TRIM27 at S173, decreased its binding with USP7, and inhibited USP7‐mediated TRIM27 deubiquitination to promote TRIM27 degradation.

Taken together, our findings reveal a USP7/TRIM27‐IKK double negative feedback loop in IECs during inflammation (Figure 8), which indicates the anti‐inflammatory function of epithelial TRIM27 and offers a new possibility for the replenishment of TRIM27 to attenuate intestinal inflammation.

## 4 Methods

### 4.1 Cell Lines and Reagents

The human differentiated colorectal adenocarcinoma cell line HT29, the human normal intestinal epithelial cell line HIEC‐6, and HEK293T cells were purchased from the American Type Culture Collection (Manassas, VA, USA). HT29 and HEK293T cells were cultured in DMEM supplemented with 10% fetal bovine serum, streptomycin and penicillin (New York, Gibco, USA) at 37°C in 5% CO_2_. RPMI 1640 medium supplemented with 10% fetal bovine serum, streptomycin and penicillin (New York, Gibco, USA) was used for HIEC‐6 cell culture.

The USP7 inhibitor P5091, the proteasome inhibitor MG132 and the protein synthesis inhibitor cycloheximide were purchased from Selleck Chemicals and Sigma‒Aldrich. The antibodies used in this study were as follows: anti‐TRIM27 (12205‐1‐AP, Proteintech, 1:1000 for WB, 1:500 for IP and 1:100 for immunohistochemistry (IHC)), anti‐USP7 (66514‐1‐Ig, Proteintech, 1:1000 for WB), anti‐TRAF6 (A23385, ABclonal, dilution 1:1000 for WB and 1:500 for IP), anti‐IKKα (A2062, ABclonal, dilution 1:1000 for WB and 1:500 for IP), anti‐IKKβ (15649‐1‐AP, Proteintech, 1:1000 for WB and IP), anti‐p‐IKKα/β (AP0546, ABclonal, dilution 1:1000 for WB), anti‐p‐IκBα (AF1870, Beyotime, dilution 1:1000 for WB), anti‐IκBα (AF1282, Beyotime, dilution 1:1000 for WB), anti‐p‐p65 (AN371, Beyotime, dilution 1:1,000 for WB), anti‐p65 (8242, Cell Signaling Technology, dilution 1:1000 for WB and 1:100 for IF), anti‐NFκB2 (15503‐1‐AP, Proteintech, dilutions: 1:1000 for WB), and anti‐MUC‐2 (ab134119, Abcam, dilution 1:100 for IF), anti‐ZO‐1 (D6L1E, Cell Signaling Technology, dilution 1:100 for IF), anti‐E‐cadherin (20874‐1‐AP, Proteintech, dilution 1:100 for IF), anti‐CK20 (A0248, Abclonal, dilution 1:100 for IF), anti‐HA (3724, Cell Signaling Technology, 1:1000 for WB), anti‐FLAG (14793, Cell Signaling Technology, 1:1000 for WB), anti‐MYC (2276, Cell Signaling Technology, 1:1000 for WB), anti‐β‐actin (A2228, Sigma‒Aldrich, 1:10 000 for WB) and anti‐GFP (sc‐8334, Santa Cruz Biotechnology, 1:1000 for WB). Normal rabbit IgG (2729, Cell Signaling Technology, for IP) was also used.

The vectors used in this study were constructed using the ClonExpress I One Step Cloning Kit (Vazyme, China). The HA‐Ub, HA‐Ub‐K48R, and HA‐Ub‐K63R plasmids were kindly provided by Dr. Wei Yu from Fudan University. The expression mutant plasmids were generated using a KOD mutagenesis kit (Toyobo, Osaka, Japan) according to the manufacturer's instructions.

### 4.2 Human Tissue Samples

Paired normal and inflamed colon tissues were collected from UC patients who underwent colorectal surgery at the Department of Colorectal Surgery, Xinhua Hospital, Shanghai Jiaotong University School of Medicine. Normal and inflamed colonic mucosal biopsies from patients with partial remission or without clinical remission after IFX treatment were collected from active patients who underwent endoscopy at the Department of Gastroenterology, Shanghai Tenth People's Hospital, Tongji University. The study was approved by the Ethics Committee of Xinhua Hospital (No. XHEC‐NSFC‐2022‐113.) The MES scoring system was used to evaluate endoscopic severity in this study. The MES used in the study indicated the result of the first colonoscopy after hospitalization. According to previous research, the MES can be classified into four (0–3) categories on the basis of endoscopic findings, such as erosions, vascular patterns, erythema, friability, and ulceration [48].

### 4.3 Mice and DSS‐induced Mice Colitis Models

*Trim27^flox/flox^* mice and *Villin‐Cre* mice with a C57BL/6 background were purchased from Cyagen Biosciences Center (Stock No: S‐CKO‐04783) and Jackson Laboratories (Stock No: 004586), respectively. The mice were backcrossed to generate *Villin‐Trim27^flox/^* ^flox^ mice, and the genotypes of the mice were determined by the PCR analysis using the genomic DNA obtained from tail biopsies. In this study, the *WT* mice we used to induce colitis were sex‐matched, age‐matched, body weight‐matched, and co‐housed littermates of the *Villin‐Trim27^flox/^* ^flox^ mice. To induce colitis, 8‐week‐old male mice (20–24 g) were fed nearly 3.0% DSS in the drinking water and were monitored for their body weight, activity, and changes in stool characteristics. The mice were sacrificed after seven consecutive days of induction, and RNA and proteins were extracted from the colon of each mouse, after which the samples were fixed in 4% paraformaldehyde for further paraffin embedding. All animal experimental procedures were approved by the Laboratory Animal Care and Welfare Committee of Xinhua Hospital affiliated with Shanghai Jiaotong University School of Medicine (No. XHEC‐F‐2025‐016).

### 4.4 Disease Activity Index (DAI) and Histological Pathological Scoring

The DAI includes body weight, stool characteristics and the degree of rectal bleeding, which are scored on a scale of 0–4 [49]. The total scores were then divided by 3 to obtain the DAI score of each mouse. Histological pathological scores were evaluated according to neutrophil infiltration, crypts, cross‐section involvement, and erosion or ulceration formation in hematoxylin–eosin‐stained slides of mouse intestines [50]. In this study, the pathological evaluation of DSS‐induced mice colitis model was conducted by two independent pathology specialists who were blinded to our research.

### 4.5 Immunohistochemistry (IHC) and Immunofluorescence (IF)

Immunohistochemical staining was performed according to a standard protocol via the heat‐induced epitope retrieval method. TRIM27 expression levels in intestinal epithelial cells were semiquantitatively evaluated by two independent pathologists who were blinded to this research. The final scores were calculated by multiplying the proportion score by the intensity score [51]. In this study, clinical patient samples with a staining score of ≤ 4 were considered to have low TRIM27 expression, and those with a score of > 4 were considered to have high TRIM27 expression.

For IF staining, SABC Cy3‐conjugated anti‐rabbit IgG was used as a secondary antibody. Finally, the samples were counterstained for nuclei with hematoxylin solution for IHC and with DAPI for IF and mounted with a coverslip. For cell immunofluorescence staining, cells grown on cover slides were fixed with 4% paraformaldehyde and permeabilized by exposure to 0.3% Triton X‐100. Then, the slides were blocked with 5% bovine serum albumin and incubated with an anti‐NF‐κB p65 mAb (1:100) overnight. AlexaFluor 594‐conjugated donkey anti‐rabbit IgG (Life Technologies, Grand Island, NY, USA) was used as the secondary antibody. The slides were then counterstained with DAPI and analyzed using fluorescence microscopy.

### 4.6 Quantitative Real‐Time PCR (qRT‒PCR)

Total RNA was extracted from tissues or cells using TRIzol reagent (Takara, Japan). Reverse transcription was performed using a PrimeScript RT Master Mix Kit (Takara, Japan). SYBR Premix ExTaq (Yeasen, Shanghai, China) and an Applied Biosystems 7500 Fast Real‐Time PCR system were used for the qPCR analysis. The relative mRNA expression was normalized to that of β‐actin and calculated by the 2^−ΔΔCt^ method. All reactions were performed in triplicate. The detailed PCR sequences of primers used in this study were listed in Table S2.

### 4.7 Immunoblotting, Immunoprecipitation and Ubiquitination Assays

For direct western blot analysis, the cells were lysed with 1% NP40 lysis buffer supplemented with NaF, Na_3_VO_4_, and a protease inhibitor cocktail (Roche). Equal amounts of protein were separated by SDS‒PAGE and transferred onto nitrocellulose membranes (Millipore, USA). Following blocking with 5% nonfat milk for 1 h at room temperature, the membrane was incubated with a specific antibody at 4°C overnight on a rotator. The specific protein was visualized with an HRP‐conjugated secondary antibody and enhanced chemiluminescence. For regular immunoprecipitation, the cells were lysed with 0.3% NP40 lysis buffer and then subjected to immunoprecipitation by incubation with anti‐FLAG/HA magnetic beads (Bimake) for 3 h at 4°C. For endogenous immunoprecipitation, the cell lysate was incubated with the indicated antibodies and control IgG conjugated with protein A/G agarose overnight. The precipitated protein was subsequently eluted from the beads, boiled with 1 × loading buffer for 10 min and analyzed by immunoblotting.

To detect ubiquitinated TRIM27 in cell lysates, HEK293 cells were transfected with HA‐Ub and the other indicated plasmids together with FLAG‐TRIM27 as indicated for 36 h. Before detecting ubiquitination, the cells were pretreated with MG132 (10 µmol L^−1^) for 6 h before lysis for subsequent analysis. The ubiquitination level of TRIM27 was determined by immunoprecipitation with an anti‐FLAG antibody followed by western blot analysis with an anti‐HA antibody. Semi‐quantitative analysis of WB was performed by analyzing the grayscale value of each band. The relative protein levels were normalized by the grayscale value of β‐actin or FLAG‐GAPDH control bands, and shown above the corresponding bands.

### 4.8 Statistical Analysis

SPSS version 22.0 (IBM 2010, Chicago, IL, USA) and GraphPad Prism 8.0 (San Diego, CA, USA) were used for statistical analysis. The quantitative data are presented as the means ± standard deviations (SDs). Two‐tailed unpaired Student's *t* tests were used to analyze the significance of two‐group comparisons. One‐way ANOVA was used to assess the statistical significance of experiments with >2 independent groups. Tukey's test was used as the post‐hoc test for the pair‐wise comparisons in one‐way ANOVA analysis. For DAI assessments, two‐way ANOVA was performed to assess statistical significance. Spearman's correlation test was used to determine the relationship between the histological pathological score and TRIM27 expression in UC patients. All the statistical tests were two‐sided and considered significant when the *p* value was <0.05.

## Author Contributions

**Peng Du**, **Chen‐Ying Liu**, and **Weimin Xu** designed the research. **Weimin Xu**, **Zhebin Hua**, and **Zhujiang Dai** performed experiments and/or analyzed data. **Weimin Xu** and **Chen‐Ying Liu** wrote the manuscript. Shasha Zhang and Yihan Jiang assisted some analysis and collected the specimens from patients. **Wensong Ge**, **YingWei Chen**, **Zhongchuan Wang**, and **Bing Zhang** collected the clinical characteristics of patients and reviewed the manuscript. All authors approved the final version.

## Funding

This work was supported by the National Natural Science Foundation of China (No. 82570638, 82470549, 82270549, 82372648), Natural Science Foundation of Shanghai (No. 25ZR1402360, 22ZR1440500), the Clinical Research Special Project of Shanghai Municipal Health Commission (20254Y0118), and the Interdisciplinary Program of Shanghai Jiaotong University (YG2025QNB40).

## Conflicts of Interest

The authors declare no conflicts of interest.

## Supporting information




**Supporting File 1**: advs75423‐sup‐0001‐SuppMat.docx.

**Supporting File 2**: advs75423‐sup‐0002‐TableS1.xlsx.

## Data Availability

The raw data supporting the conclusions of this article will be made available by the authors, without undue reservation.
