# Supplementary material for: Epithelial TRIM27 Inhibits Intestinal Inflammation in Ulcerative Colitis by the USP7/TRIM27‐IKK Double Negative‐Feedback
Source: Adv Sci (Weinh). 2026 Apr 22;13(40):e22114. doi: 10.1002/advs.202522114 (PMC13335431; doi:10.1002/advs.202522114)
Supplement: Supplementary file 1 — Supporting File 1: advs75423‐sup‐0001‐SuppMat.docx. [file ADVS-13-e22114-s001.docx]

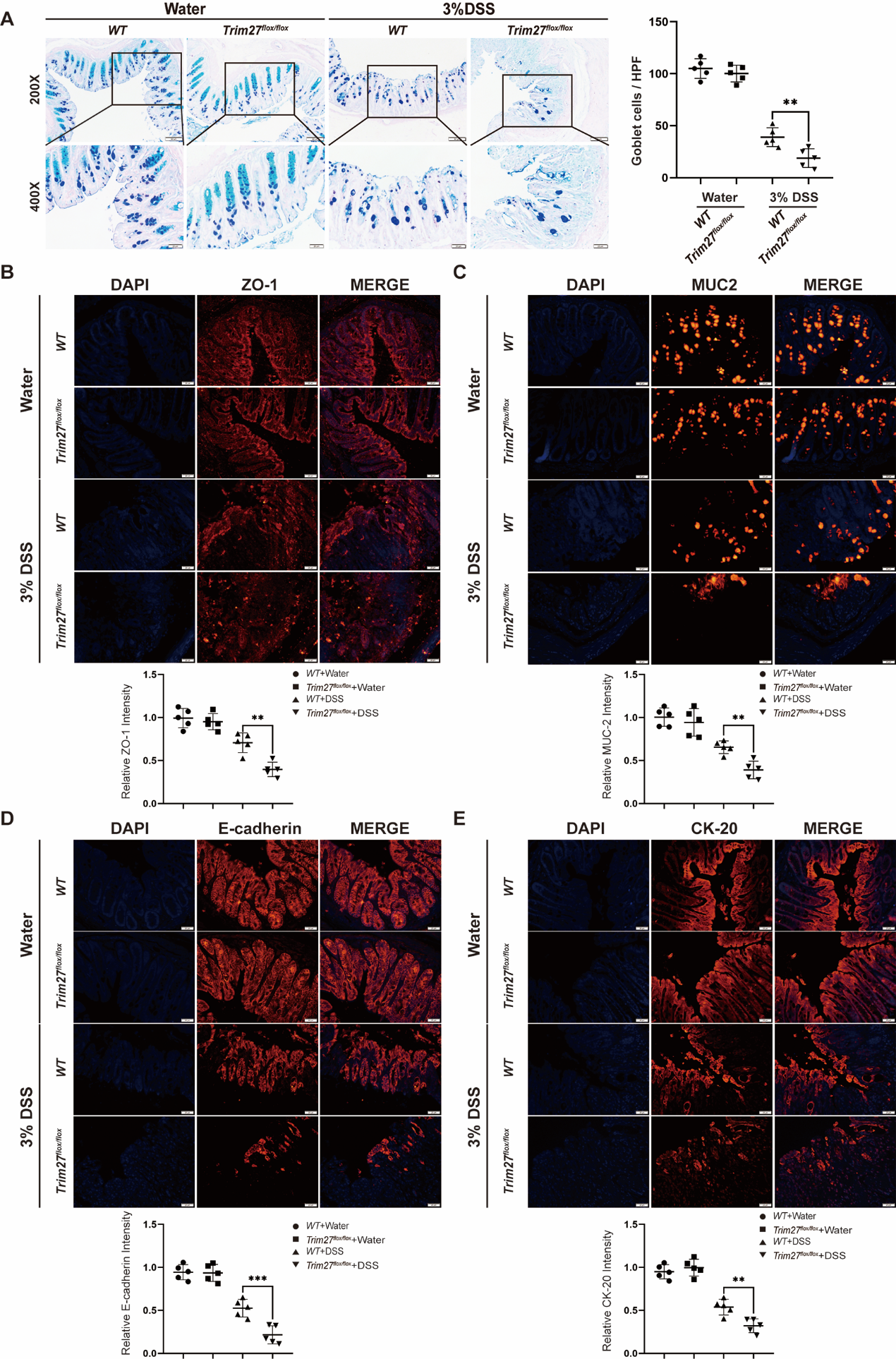


**Supplementary Figure 1. TRIM27 knockdown aggravates DSS-induced intestinal mucosal barrier damage in mice.** (A) Alcian blue/periodic acid-Schiff staining of goblet cells in colons from control and DSS-treated *WT* and *Villin-Trim27^flox/flox^* mice (magnification: 200×, upper panels; 400×, lower panels. (B-E) Representative images of ZO-1 (B), MUC2 (C), E-cadherin (D) and CK20 (E) immunofluorescence in control and DSS-treated *WT* and *Villin-Trim27^flox/flox^* mice (magnification: 200×; upper panels; 400×; lower panels). The data are presented as the means ± SDs. n=5 biologically independent samples for the control group; n=5 biologically independent samples for the DSS group (A). One-way ANOVA (A-E) was performed to assess statistical significance. ** *P* < 0.01, *** *P* < 0.001.


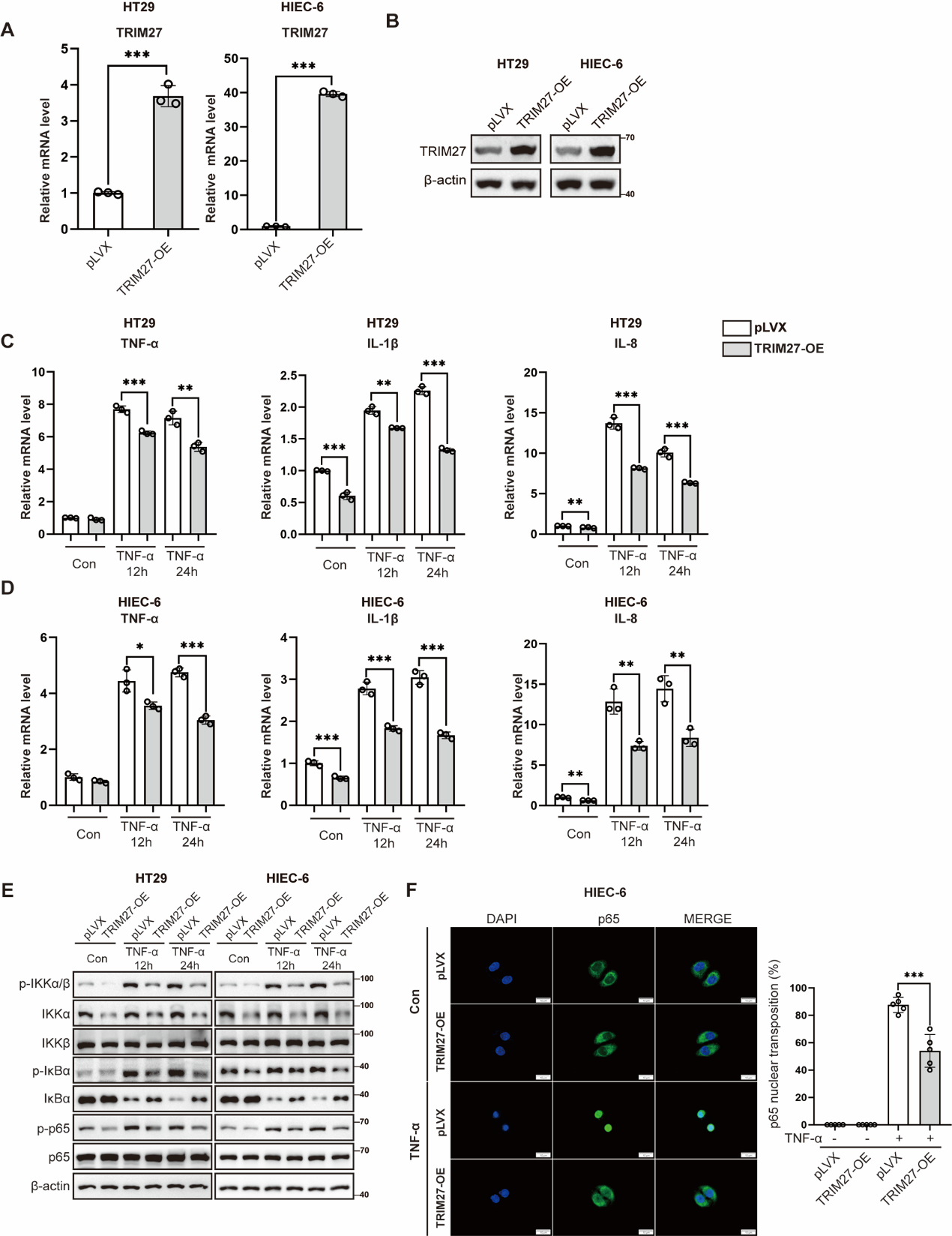


**Supplementary Figure 2. Enforced expression of TRIM27 inhibits the TNF-α-induced inflammatory response in IECs.** (A-B) The efficiency of TRIM27 overexpression was examined in HT29 and HIEC-6 cells by qRT‒PCR (A) and western blot analysis (B). (C-D) The mRNA levels of proinflammatory cytokines were measured by qRT‒PCR in TRIM27-overexpressing and control HT29 (C) and HIEC-6 (D) cells after exposure to TNF-α (10 ng/ml) for the indicated times. (E) Immunoblotting was performed to measure the levels of p-IKKα/β, IKKα, IKKβ, p-IκBα, IκBα, p-p65 and p65 in control and TRIM27-overexpressing HT29 and HIEC-6 cells after TNFα stimulation for the indicated times. (F) Immunofluorescence staining was performed to assess the nuclear localization of p65 in control and TRIM27-overexpressing HIEC-6 cells after TNF-α treatment (10 ng/ml) for 2 h. The data are presented as the means ± SDs. n=3 (A, C, D) biologically independent samples per group. The data (A, C, D) are representative of 3 independent experiments. Two-tailed, unpaired Student’s t test (A, B) and One-way ANOVA (C, D, F) were performed to assess statistical significance. * *P* < 0.05, ** *P* < 0.01, *** *P* < 0.001.


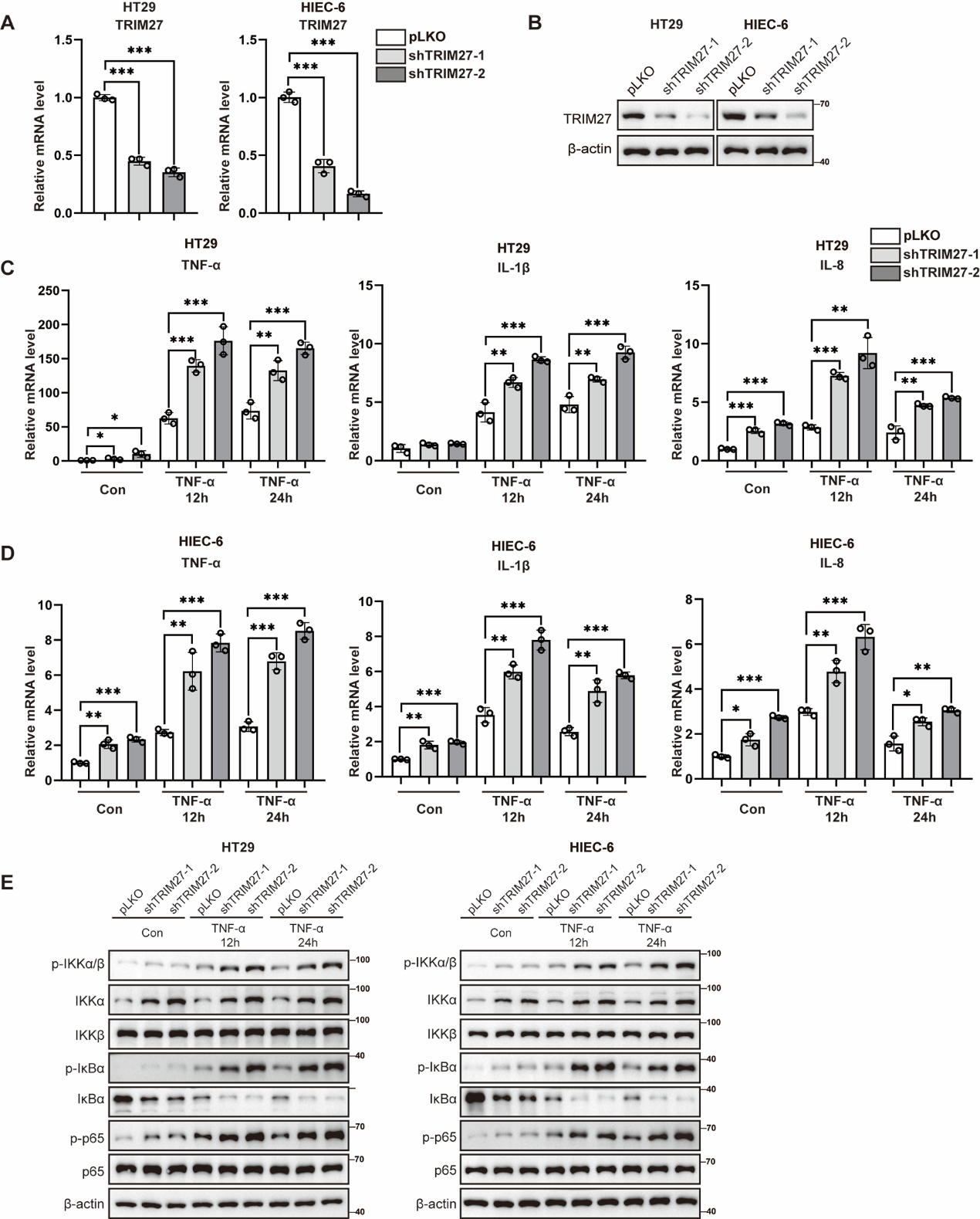


**Supplementary Figure 3. TRIM27 knockdown promotes TNF-α induced intestinal inflammatory response.** (A-B) The efficiency of TRIM27 knockdown was examined in HT29 and HIEC-6 cells by qRT‐PCR and western blot analysis. (C-D) Expression of proinflammatory cytokines were examined by qRT-PCR in TRIM27-delepted and control HT29 and HIEC-6 cells after exposed to TNF-α (10 ng/ml) for indicated times. (E) Immunoblotting was performed to measure the levels of p-IKKα/β, IKKα, IKKβ, p-IκBα, IκBα, p-p65 and p65 in control and TRIM27-knockdown HT29 and HIEC-6 cells after TNFα stimulation for the indicated times. The data (A, C, D) are representative of 3 independent experiments. One-way ANOVA (A, C, D) were performed to assess statistical significance. * *P* < 0.05, ** *P* < 0.01, *** *P* < 0.001.


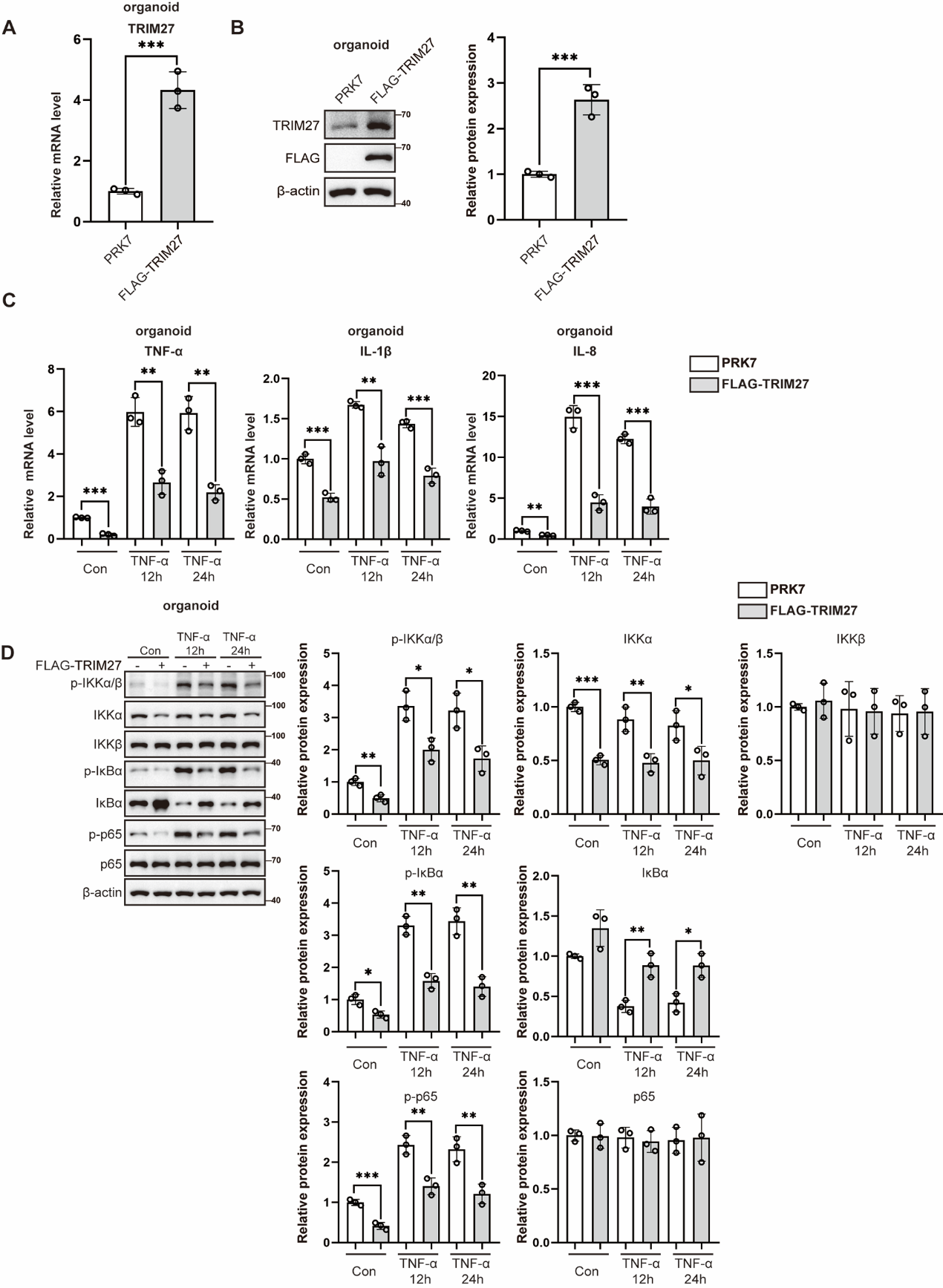


**Supplementary Figure 4. Enforced expression of TRIM27 inhibits the TNF-α-induced inflammatory response in human colon organoids.** (A-B) The efficiency of TRIM27 overexpression was examined in colon organoids by qRT‒PCR (A) and western blot analysis (B). (C) The mRNA levels of proinflammatory cytokines were measured by qRT‒PCR in TRIM27-overexpressing and control colon organoids after exposure to TNF-α (10 ng/ml) for the indicated times. (D) Immunoblotting was performed to measure the levels of p-IKKα/β, IKKα, IKKβ, p-IκBα, IκBα, p-p65 and p65 in control and TRIM27-overexpressing colon organoids after TNFα stimulation for the indicated times. The data are presented as the means ± SDs. n=3 (A, C) biologically independent samples per group. Two-tailed, unpaired Student’s t test (A, B) and One-way ANOVA (C, D) were performed to assess statistical significance. * *P* < 0.05, ** *P* < 0.01, *** *P* < 0.001.


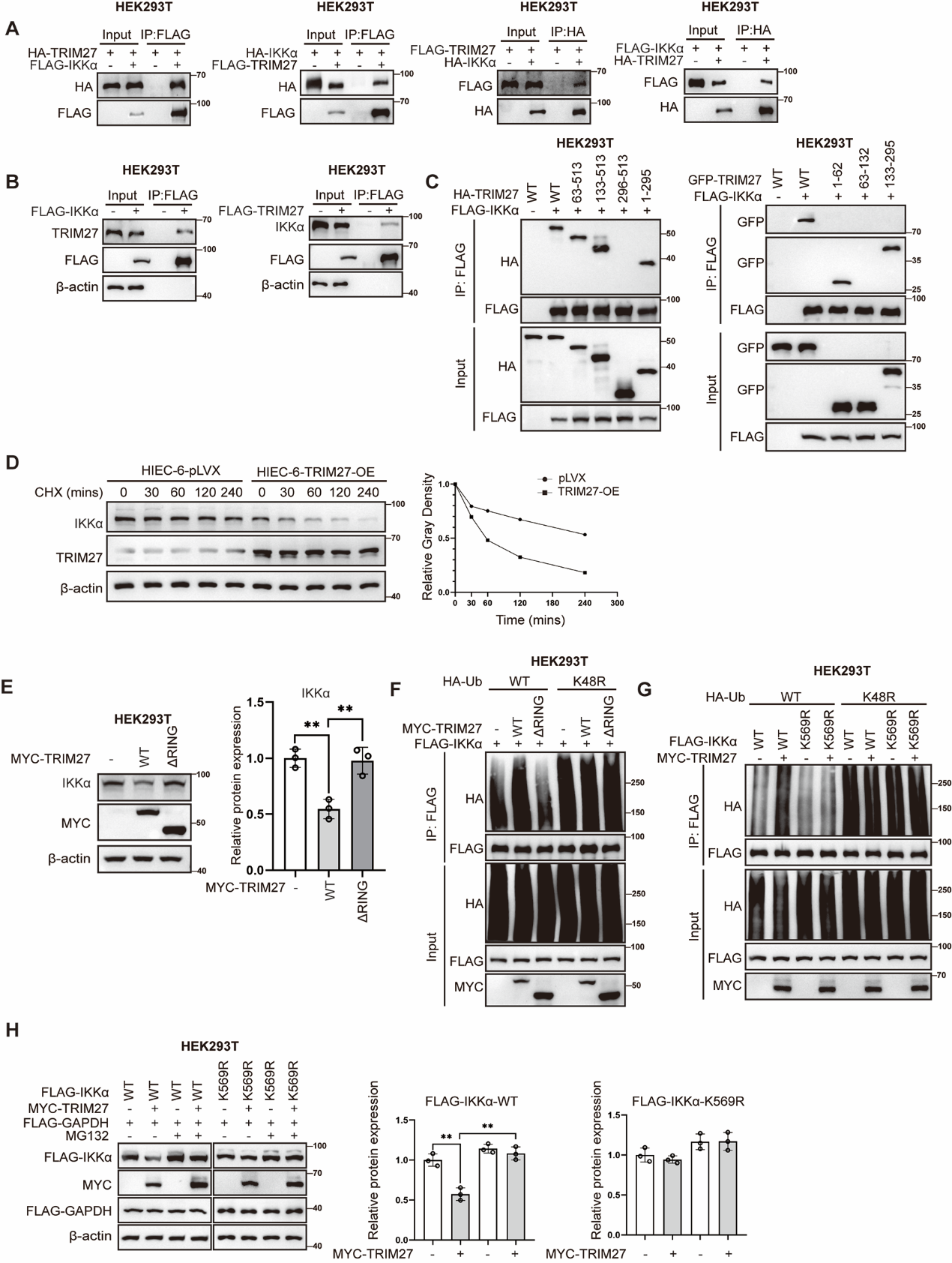


**Supplementary Figure 5. TRIM27 promotes the K48-linked ubiquitylation of IKKα.** (A) Exogenous co-IP of FLAG-IKKα and HA-TRIM27, or FLAG-TRIM27 and HA- IKKα in HEK293T cells. (B) Semiendogenous co-IP of exogenous FLAG-IKKα and endogenous TRIM27 and exogenous FLAG-TRIM27 and endogenous IKKα in HEK293T cells. (C) Exogenous co-IP of FLAG-IKKα and full-length/truncated HA or GFP-TRIM27 in HEK293T cells. (D) Analysis of the IKKα protein half-life in control and TRIM27-overexpressing HIEC-6 cells. The cells were treated with cycloheximide (CHX) for the indicated times before western blot analysis of IKKα expression. (E) HEK293T cells were transfected with the indicated control, MYC-TRIM27^WT^, and MYC-TRIM27^ΔRING^ plasmids. IKKα expression was determined via western blotting. (F) HEK293T cells were transfected with HA-Ub, HA-Ub-K48R, MYC-TRIM27^WT^, MYC-TRIM27^ΔRING^ or FLAG-IKKα. FLAG-IKKα was immunoprecipitated, and its ubiquitination level was measured via western blotting. (G) HEK293T cells were transfected with HA-Ub, HA-Ub-K48R, FLAG- IKKα^WT^, FLAG- IKKα^K569R^, or MYC-TRIM27. The ubiquitination level was analyzed as described above. (H) The protein levels of the WT and K569R mutant FLAG-IKKα were detected in HEK293T cells transfected with control and MYC-TRIM27 plasmids with or without MG132 treatment. One-way ANOVA (E, H) was performed to assess statistical significance. ** *P* < 0.01.


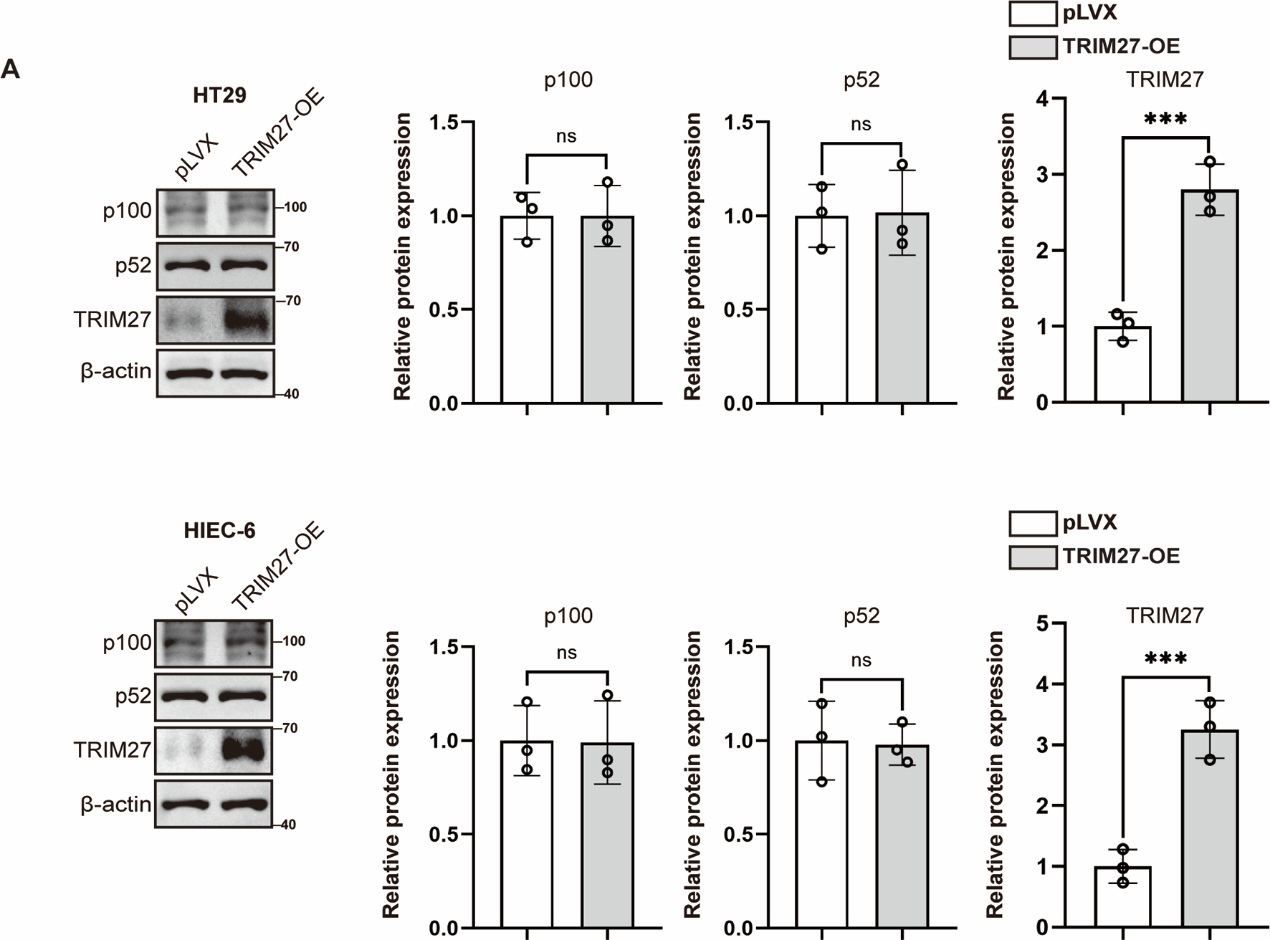


**Supplementary Figure 6. TRIM27 failed to affect the non-canonical NF-κB pathway.** (A) The protein expression of p100 and p52 were detected in TRIM27-overexpressing and control HT29 and HIEC-6 cells by western blot analysis. Unpaired Student’s t test was performed to assess statistical significance. *** *P* < 0.001.


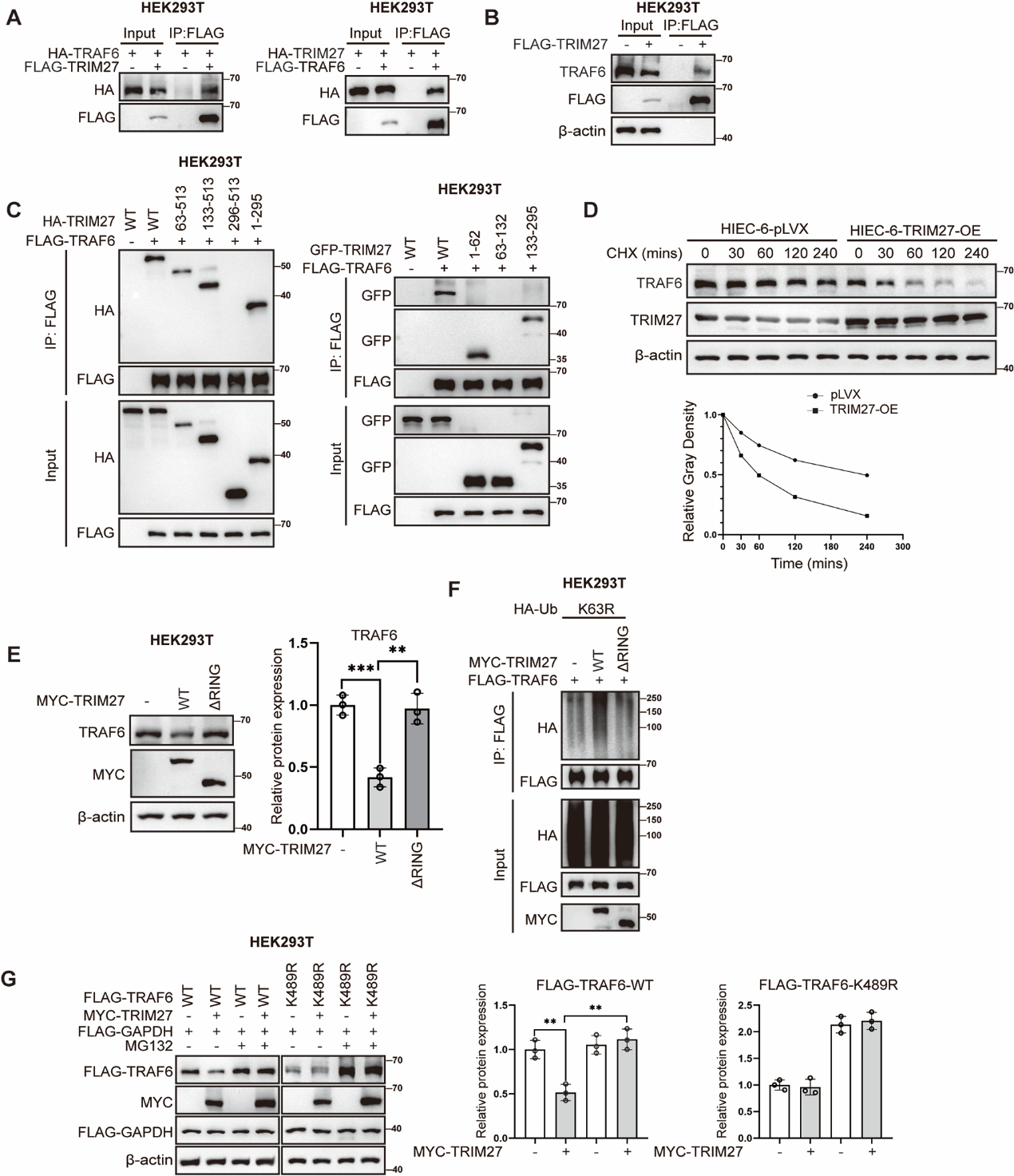


**Supplementary Figure 7. TRIM27 promotes the K48-linked ubiquitylation of TRAF6.** (A) Exogenous co-IP of FLAG-TRIM27 and HA-TRAF6, or FLAG-TRAF6 and HA-TRIM27 in HEK293T cells. (B) Semiendogenous co-IP of exogenous FLAG- TRIM27 and endogenous TRAF6 in HEK293T cells. (C) Exogenous co-IP of FLAG-TRAF6 and full-length/truncated HA or GFP-TRIM27 in HEK293T cells. (D) Analysis of the TRAF6 protein half-life in control and TRIM27-overexpressing HIEC-6 cells. The cells were treated with CHX (75 μg/ml) for the indicated times before western blot analysis of TRAF6 expression. (E) HEK293T cells were transfected with the indicated control, MYC-TRIM27^WT^, and MYC-TRIM27^ΔRING^ plasmids. TRAF6 expression was determined via western blotting. (F) HEK293T cells were transfected with HA-Ub-K63R, MYC-TRIM27^WT^, MYC-TRIM27^ΔRING^ or FLAG-TRAF6. FLAG-TRAF6 was immunoprecipitated, and its ubiquitination level was measured via western blotting. (G) The protein levels of the WT and K489R mutant FLAG-TRAF6 were detected in HEK293T cells transfected with control and MYC-TRIM27 plasmids with or without MG132 treatment. One-way ANOVA (E, G) was performed to assess statistical significance. ** *P* < 0.01, *** *P* < 0.001.


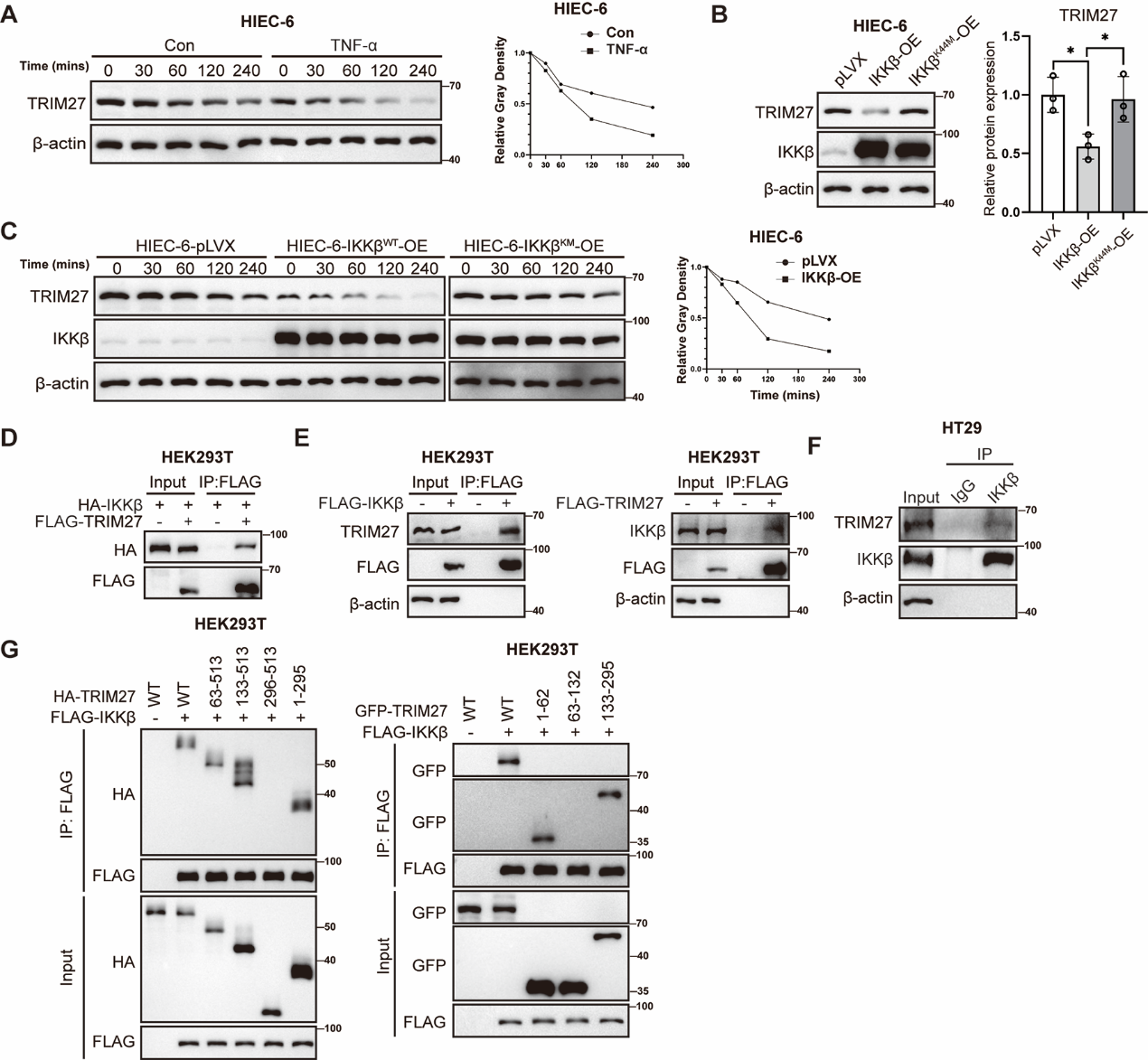


**Supplementary Figure 8. IKKβ inhibits the stabilization of the TRIM27 protein.** (A) Analysis of the half-life of the TRIM27 protein in HIEC-6 cells with or without TNF-α (10 ng/ml) treatment. The cells were treated with CHX (75 μg/ml) for the indicated times before western blot analysis of TRIM27 expression. (B) Western blot analysis of TRIM27 expression in HIEC-6 cells stably overexpressing IKKβ^WT^ or IKKβ^K44M^ and the corresponding control cells. (C) Analysis of the TRIM27 protein half-life in control and IKKβ^WT^ or IKKβ^K44M^-overexpressing HIEC-6 cells. Cells were treated with CHX (75 μg/ml) for the indicated times before western blot analysis of TRIM27 expression. (D) Exogenous co-IP of HA-IKKβ and FLAG-TRIM27 in HEK293T cells. (E) Semiendogenous co-IP of exogenous FLAG-IKKβ and endogenous TRIM27, and exogenous FLAG-TRIM27 and endogenous IKKβ. (F) Endogenous Co-IP of TRIM27 and IKKβ in HT29 cells. (G) Exogenous co-IP of FLAG-IKKβ and full-length/truncated HA or GFP-TRIM27 in HEK293T cells. One-way ANOVA (B) was performed to assess statistical significance. * *P* < 0.05.


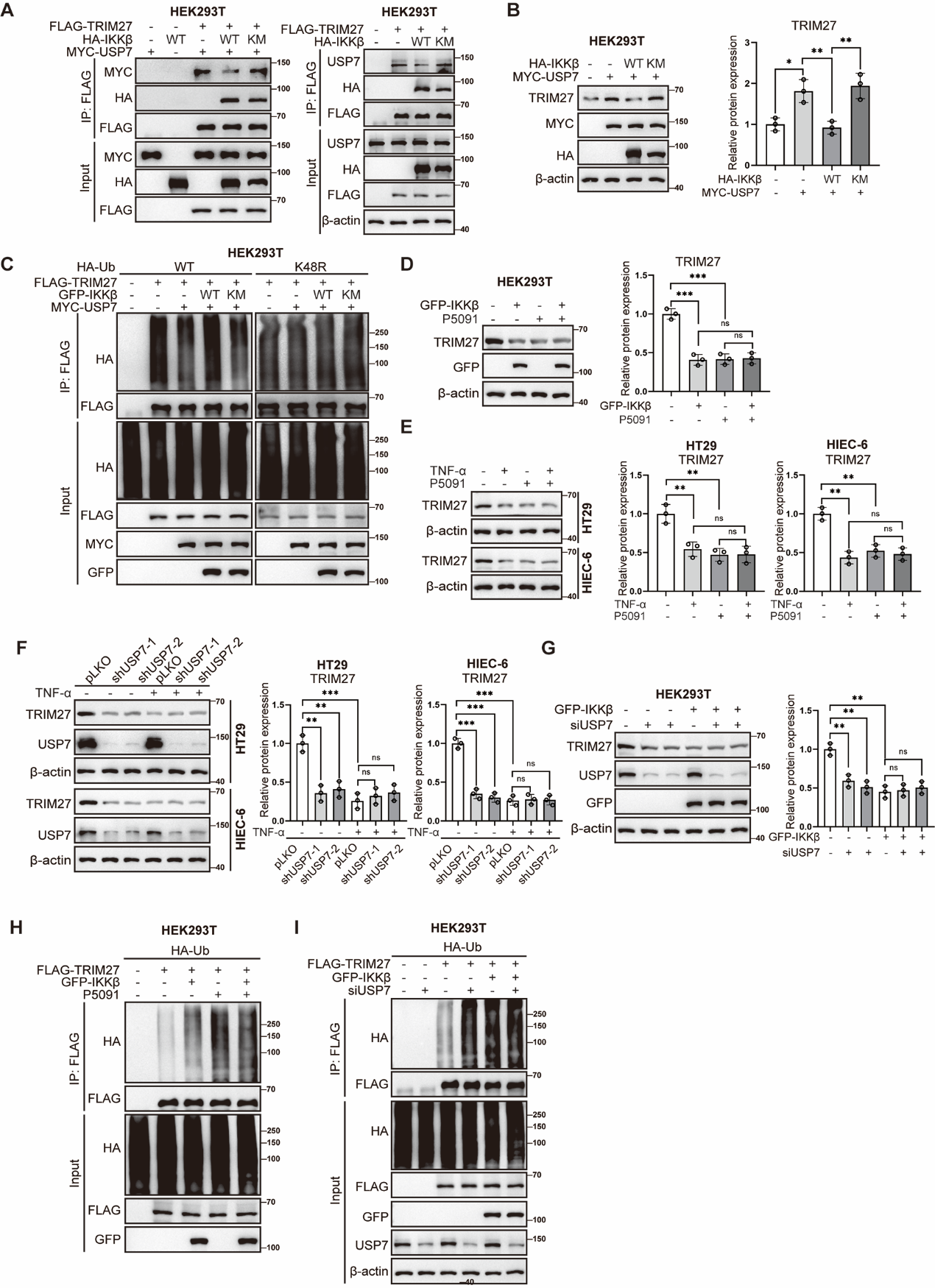


**Supplementary Figure 9. IKKβ inhibits the interaction between TRIM27 and USP7 by its kinase activity.** (A) Exogenous co-IP of FLAG-TRIM27 and MYC-USP7 in HEK293T cells coexpressing HA-IKKβ^WT^ or HA-IKKβ^K44M^. Semiendogenous co-IP of exogenous FLAG-TRIM27 and endogenous USP7 in HEK293T cells coexpressing HA-IKKβ^WT^ or HA-IKKβ^K44M^. (B) HEK293T cells were transfected with the indicated control vector, MYC-USP7, HA-IKKβ^WT^ or HA-IKKβ^K44M^ plasmids. The cells were lysed, and TRIM27 expression was measured by western blotting. (C) HEK293T cells were transfected with the indicated HA-Ub, HA-Ub-K48R, FLAG-TRIM27, MYC-USP7, GFP-IKKβ^WT^ or GFP-IKKβ^K44M^ plasmids. The cell lysates were used for immunoprecipitation with an anti-FLAG antibody for subsequent quantification of TRIM27 ubiquitination by western blotting. (D) Western blot analysis of TRIM27 expression in HEK293T cells transfected with the control vector, or with GFP-IKKβ with or without P5091 (10 μM) treatment. (E) Western blot analysis of TRIM27 expression in HT29 and HIEC-6 cells treated with TNF-α (10 ng/ml) alone or in combination with P5091 (10 μM) for 24h. (F) Western blot analysis of TRIM27 expression in USP7-knockdown and control HT29 and HIEC-6 cells with or without TNF-α (10 ng/ml) treatment for 12h. (G) HEK293T cells were transfected with control or siRNA targeting USP7 and then transfected with GFP-IKKβ. TRIM27 expression was determined via western blotting. (H) HEK293T cells were transfected with HA-Ub or FLAG-TRIM27 plasmids with or without P5091 (10 μM) treatment. FLAG-TRIM27 was immunoprecipitated, and its ubiquitination level was measured via western blotting. (I) Control and USP7-deleted HEK293T cells were transfected with HA-Ub, FLAG-TRIM27 and GFP-IKKβ plasmids. FLAG-TRIM27 was immunoprecipitated for subsequent quantification of the ubiquitination level of TRIM27 by western blots. One-way ANOVA (B, D, E, F, G) was performed to assess statistical significance. * *P* < 0.05, ** *P* < 0.01, *** *P* < 0.001.


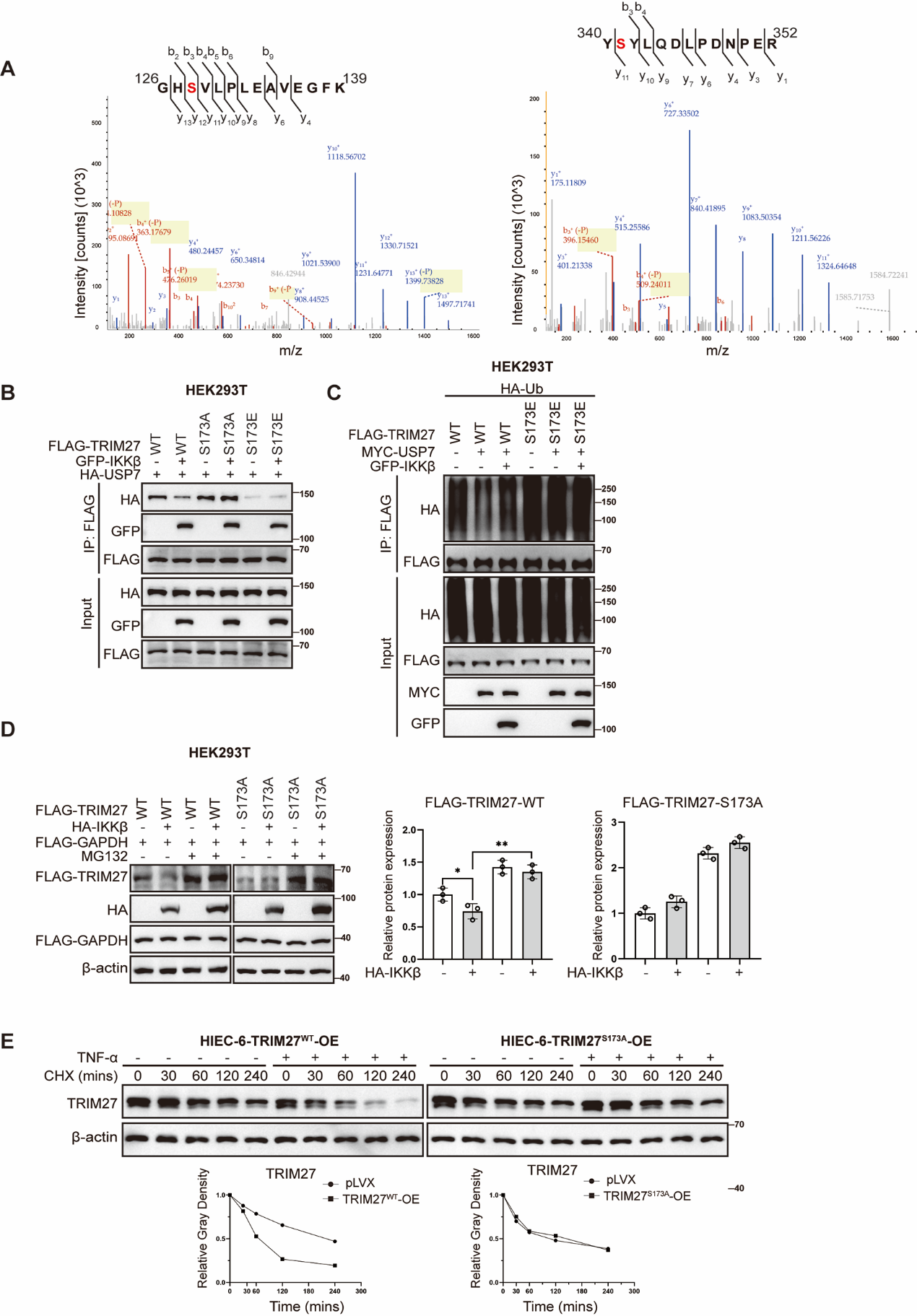


**Supplementary Figure 10. S173-TRIM27 phosphorylation by IKKβ kinase decreases the binding with USP7**. (A) Identification of phosphorylated TRIM27-S128 and TRIM27-S341 in control and IKKβ-overexpressing HEK293T cells with transfection of FLAG-TRIM27 by tandem mass spectrometry. (B) Exogenous co-IP of HA-USP7 and WT, S173A, and S173E mutants of FLAG-TRIM27 with or without the transfection of GFP-IKKβ. (C) HEK293T cells were transfected with the indicated HA-Ub, MYC-USP7, GFP-IKKβ, WT and S173E FLAG-TRIM27 plasmids. The cell lysates were used for immunoprecipitation with an anti-FLAG antibody for subsequent quantification of TRIM27 ubiquitination by western blotting. (D) The protein levels of the WT and S173A mutant FLAG-TRIM27 were detected in HEK293T cells transfected with control and HA-IKKβ plasmids with or without MG132 treatment. (E) Analysis of the TRIM27 protein half-life in control and TRIM27^WT^ or TRIM27^S173A^ overexpressing HIEC-6 cells with or without TNF-α (10 ng/ml) treatment. The cells were treated with CHX (75 μg/ml) for the indicated times before western blot analysis of TRIM27 expression. One-way ANOVA (D) was performed to assess statistical significance. * *P* < 0.05, ** *P* < 0.01.

| **Supplementary Table 2: The primers and shRNA sequence used in this study.** | | |
| --- | --- | --- |
| **Primers** | **Sequence** |  |
| TRIM27 (human) | Forward: AGCCCATGATGCTCGACTG | |
|  | Reverse: GGGCACGACACGTTAGTCT | |
| TNF-α (human) | Forward: CCTCTCTCTAATCAGCCCTCTG | |
|  | Reverse: GAGGACCTGGGAGTAGATGAG | |
| IL-6 (human) | Forward: ACTCACCTCTTCAGAACGAATTG | |
|  | Reverse: CCATCTTTGGAAGGTTCAGGTTG | |
| IL-1β (human) | Forward: ATGATGGCTTATTACAGTGGCAA | |
|  | Reverse: GTCGGAGATTCGTAGCTGGA | |
| IL-8 (human) | Forward: ACTCCAAACCTTTCCACC | |
|  | Reverse: CTTCTCCACAACCCTCTG | |
| IL-17A (human) | Forward: TCCCACGAAATCCAGGATGC | |
|  | Reverse: GGATGTTCAGGTTGACCATCAC | |
| β-actin (human) | Forward: CATGTACGTTGCTATCCAGGC | |
|  | Reverse: CTCCTTAATGTCACGCACGAT | |
| TNF-α (mouse) | Forward: CAGGCGGTGCCTATGTCTC | |
|  | Reverse: CGATCACCCCGAAGTTCAGTAG | |
| IL-6 (mouse) | Forward: TAGTCCTTCCTACCCCAATTTCC | |
|  | Reverse: TTGGTCCTTAGCCACTCCTTC | |
| IL-1β (mouse) | Forward: GAAATGCCACCTTTTGACAGTG | |
|  | Reverse: TGGATGCTCTCATCAGGACAG | |
| GAPDH (mouse) | Forward: AGGTCGGTGTGAACGGATTTG | |
|  | Reverse: GGGGTCGTTGATGGCAACA | |
